# Pyrethroid exposure alters internal and cuticle surface bacterial communities in *Anopheles albimanus*

**DOI:** 10.1038/s41396-019-0445-5

**Published:** 2019-06-06

**Authors:** Nsa Dada, Juan C. Lol, Ana Cristina Benedict, Francisco López, Mili Sheth, Nicole Dzuris, Norma Padilla, Audrey Lenhart

**Affiliations:** 1 Entomology Branch, Division of Parasitic Diseases and Malaria, Center for Global Health, United States Centers for Disease Control and Prevention, Atlanta, GA USA; 2 American Society for Microbiology, Washington, DC USA; 3 Grupo de Biología y Control de Vectores, Centro de Estudios en Salud, Universidad del Valle de Guatemala, Guatemala, Guatemala; 4 Biotechnology Core Facility Branch, Division of Scientific Resources, National Center for Emerging & Zoonotic Infectious Diseases, United States Centers for Disease Control and Prevention, Atlanta, GA USA

**Keywords:** Molecular biology, Animal physiology, Microbial ecology, Metagenomics, Microbiome

## Abstract

A deeper understanding of the mechanisms underlying insecticide resistance is needed to mitigate its threat to malaria vector control. Following previously identified associations between mosquito microbiota and insecticide resistance, we demonstrate for the first time, the effects of pyrethroid exposure on the microbiota of F_1_ progeny of field-collected *Anopheles albimanus*. Larval and adult mosquitoes were exposed to the pyrethroids alphacypermethrin (only adults), permethrin, and deltamethrin. While there were no significant differences in bacterial composition between insecticide-resistant and insecticide-susceptible mosquitoes, bacterial composition between insecticide-exposed and non-exposed mosquitoes was significantly different for alphacypermethrin and permethrin exposure. Along with other bacterial taxa not identified to species, *Pantoea agglomerans* (a known insecticide-degrading bacterial species) and *Pseudomonas fragi* were more abundant in insecticide-exposed compared to non-exposed adults, demonstrating that insecticide exposure can alter mosquito bacterial communities. We also show for the first time that the cuticle surfaces of both larval and adult *An. albimanus* harbor more diverse bacterial communities than their internal microbial niches. Together, these findings demonstrate how insecticide pressure could be selecting for certain bacteria within mosquitoes, especially insecticide-metabolizing bacteria, thus potentially contributing to insecticide resistance.

## Introduction

Recent evidence suggests that progress in global malaria control has stalled, with an estimated 2 million more malaria cases in 2017 than in 2016, and an increase in malaria incidence in the region of the Americas [1]. This stall in progress overlaps with increasing reports of insecticide resistance [1, 2], which poses a growing challenge to malaria vector control programs [3]. The mechanisms underlying insecticide resistance in malaria vectors are not fully understood. While the following four main mechanisms underlying insecticide resistance in mosquitoes have been described [4]: cuticular modifications, insecticide target site insensitivity, heightened insecticide detoxification, and behavioral avoidance of insecticides, significant gaps still remain, especially regarding the factors underlying the increasing intensity of insecticide resistance in mosquito populations. The increase in access to advanced genomic tools has now made it easier to investigate other aspects of mosquito biology, such as their microbiota, that may be associated with insecticide resistance.

Mosquitoes, like other living organisms, are hosts to a variety of microbes that are principally acquired from their breeding habitats during immature development, and from adult food sources [5]. In addition to habitat and/or food source-acquired microbes, transovarial bacterial transmission from adult females to their eggs [6] and transstadial transmission across immature stages [7] and onto the adult stage [6] have also been demonstrated in mosquitoes. These microbes, some of which are known to metabolize insecticides [8–10], actively shape their host physiology [5, 11]. The mosquito microbiota may thus contribute to insecticide detoxification, consequently augmenting resistance in the host; a phenomenon that has previously been demonstrated in agricultural pests [12–16] but only now being investigated in disease vectors [9, 17]. We previously studied the microbiota of field-caught Peruvian *An. albimanus* [9] and detected significant differences in the composition and putative functions of bacteria between fenitrothion-resistant and fenitrothion-susceptible mosquitoes. These results provided a comprehensive baseline regarding the bacterial composition of *An. albimanus* in relation to insecticide resistance and suggested that the mosquito microbiota could be affected by insecticides and/or contribute to insecticide resistance.

Malaria vector control programs in the Americas rely heavily on pyrethroid insecticides, used mainly on insecticide-treated bednets or for indoor residual spraying (IRS) [18], thus increasing pyrethroid selection pressure on malaria vector populations. However, information on the underlying mechanisms of pyrethroid resistance in malaria vectors in the Americas remain poorly characterized, despite intensified vector control efforts stemming from regional malaria elimination programs [19]. To characterize these mechanisms of pyrethroid resistance, especially from the perspective of the microbiota, the present study focuses on describing the effects of pyrethroid exposure on the microbiota of *An. albimanus* from Guatemala. We hypothesized that any effect of insecticide exposure on the mosquito microbiota would be more evident on the cuticle surface compared to the internal microbial community since the cuticle is an insect’s outermost covering and thus first point of contact with insecticides [20]. In addition, changes to the cuticle have been implicated in insecticide resistance [21]. Our objectives were to characterize and compare the composition of internal and cuticle surface bacteria between adult and larval F_1_ progeny of field-collected mosquitoes that were either exposed (and further classified as pyrethroid resistant or pyrethroid susceptible) or not exposed to pyrethroid insecticides. In order to check for spatial consistency, we tested F_1_ progeny originating from multiple locations. We discuss our findings in light of the hypothesis that mosquito microbiota is affected by insecticides and contribute to the detoxification of insecticides within the host. We also report the first comprehensive characterization of *An. albimanus* larval microbiota, as well as the mosquito cuticle surface microbiota.

## Materials and methods

### Mosquito collections and rearing

**Fig. 1:**
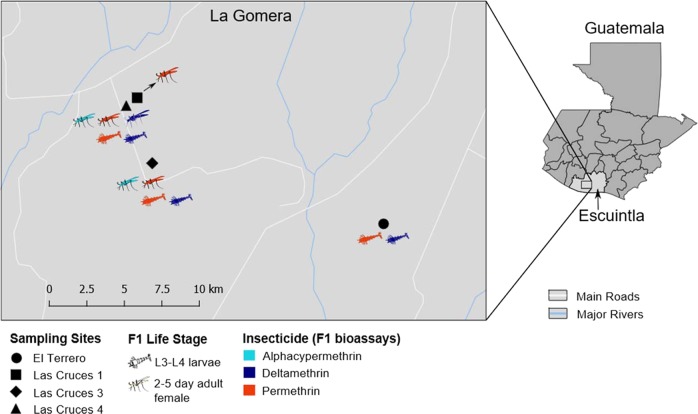
Sampling sites of field-caught *An. albimanus* and details of bioassays on resulting F_1_ progeny. This figure shows the map of Guatemala indicating the Department of Escuintla (right) and an expanded view of La Gomera Municipality indicating the four sites from which field-collected mosquitoes were sampled (left). F_1_ larvae (*n* = 132) and adult (*n* = 135) progeny originating from these sites and tested for insecticide resistance, were sequenced. For each collection site, the figure shows the developmental stage of F_1_ progeny tested, color-coded by the type of insecticide they were tested for. See Suppl. 2 for a summary of the number of larvae and adults processed for each insecticide per site

Gravid adult female *An. albimanus* were collected in and around four cattle corrals sampled in two villages: El Terrero and Las Cruces, in La Gomera, Escuintla, Guatemala (Fig. 1). Mosquito samples, separated by collection site, were held in paper cups with access to cotton pads soaked in 10% sucrose solution, and transported to the insectary at Universidad del Valle de Guatemala in Guatemala City for species identification and oviposition. Mosquitoes were morphologically sorted using identification keys [22]. Approximately 300 gravid females identified as *An. albimanus* based on the presence of a white terminal palpal segment, white third and fourth hind tarsomeres, and basal dark band on fifth hind tarsomere were used for oviposition. Species identification was confirmed by PCR (as described below) performed on DNA from the legs of a subsample of the F_1_ progeny.

Oviposition was achieved using a previously described method [23] with modifications. Briefly, oviposition containers (ovipots) were created by placing gravid females in 32 oz. paper soup containers (60 ± 10 mosquitoes/container) containing distilled water to a depth of 2–3 cm. Prior to the introduction of mosquitoes, the containers were covered with netting, subsequently topped with cotton pads soaked in 10% sucrose solution, then covered with a thick black plastic bag to trap in moisture and keep the containers dark. The ovipots were kept under standard insectary conditions of 27 ± 2 °C, 80 ± 10% relative humidity, and a 12-h light–dark cycle for at least 48 h to allow for oviposition, after which adults were removed and eggs were collected. Under the aforementioned conditions, immature mosquitoes were reared using distilled water with F_1_ progeny of parents from the same collection site grouped together and reared separately from those of parents from other sites. Eggs from ovipots were washed into 18 × 14 × 3 inch plastic larval trays (150–200 eggs per tray) containing distilled water to a depth of 2–3 cm, with 3–4 drops of 10% yeast solution. Hatched larvae were fed finely ground koi food (Foster & Smith, Inc. Rhinelander, WI) until pupation. All larvae were reared under identical conditions to eliminate any bias that could have been introduced due to dissimilar rearing conditions. Approximately a quarter of the proportion of resulting third to fourth instar larvae (L3–L4) were collected for insecticide resistance assays, and the remaining larvae were reared until the pupal stage. With the aid of a stereo microscope, male and female pupae were separated into 8 oz. paper cups placed in cardboard cages for adult emergence. Adult virgin females were provided 10% sucrose solution until they were aged 2–5 days then used for insecticide resistance assays.

### Determination of pyrethroid resistance status in larval and adult F_1_ progeny of field-collected *An. albimanus*

Three different pyrethroid insecticides were used for this study: alphacypermethrin, deltamethrin, and permethrin. Owing to a limited number of mosquitoes, adult mosquitoes were tested for resistance to all three insecticides, while larvae were tested with only deltamethrin and permethrin (Fig. 1). The diagnostic dose (a dose that kills 100% of susceptible mosquitoes within a given time) and diagnostic time (the expected time to achieve 100% mortality) [24] for each insecticide were determined (Suppl. 1A) using L3–L4 larvae from the insecticide-susceptible *An. albimanus* Sanarate strain [25]. Insecticide resistance assays were performed in the insectary under the aforementioned rearing conditions. For larvae, 100 mL of distilled water was added to 150 mL glass beakers, followed by 1 mL of insecticide stock solution. Absolute ethanol (1 mL) without added insecticide was added to the control beaker. The beakers were left to stand for 10–15 min to allow the solvent to evaporate. Subsequently, 15–20 L3–L4 larvae were added to each beaker, and mortality was scored at the end of the diagnostic time (Suppl. 1A). Larvae that were alive at the end of the assays were classified as resistant, those that were dead or moribund were classified as susceptible, while those that were used in the control beaker (all survived) were classified as non-exposed. For each insecticide tested, three replicates of the assay were run, and resistant, susceptible, and non-exposed samples from all replicates were pooled for further processing.

Using the Centers for Disease Control and Prevention (CDC) bottle bioassay method [24], the adult insecticide resistance assays were performed on adult F_1_ females of uniform physiological status. We used virgin adult females that were aged 2–5 days and non-blood fed to eliminate potential confounding effects since age and blood meal status are known to affect the mosquito microbiota [26]. Each insecticide was tested using the recommended diagnostic dose and time [24] for *Anopheles* (Suppl. 1B), and at the end of the assays, mosquitoes were classified as resistant, susceptible, or non-exposed and were immediately placed in RNALater® (Applied Biosystems, Foster City, CA). For both larvae and adults, resistant and non-exposed samples were euthanized by placing them in liquid nitrogen or at −80 °C. All samples were preserved in RNALater® and held at −80 °C until shipping (on dry ice) to the CDC in Atlanta, USA, where molecular analyses were performed.

### Extraction and purification of genomic DNA from whole larvae and adult mosquitoes and the surface of their cuticles

A total of 132 larvae and 135 adults were categorized as resistant, susceptible, or non-exposed to each tested insecticide and further processed. Prior to genomic DNA extraction, mosquito samples were thawed at 4 °C overnight, and the RNALater® solution was subsequently discarded. Samples were then rinsed once with nuclease-free water to remove any remaining RNALater® solution and pooled for further processing. Samples were grouped by parental collection site, and for each insecticide tested, three pools of mosquitoes (at both the larval and adult stages), comprising three mosquitoes per pool, were processed in each category unless otherwise stated. To obtain the microbiota from the surface of the cuticle, each pool of larvae or adult mosquitoes was washed in nuclease-free water (500 µL) by agitating with a vortex mixer for 15–20 s, and the resulting wash solution was transferred to a new tube for DNA extraction. The pools of whole larvae or adults were subsequently surface sterilized using ethanol washes as previously described [9].

Genomic DNA was extracted from pools of surface-sterilized whole larvae and adult *An. albimanus* as well as from the washed cuticle surface of each respective pool, using the DNeasy Blood and Tissue Kit (QIAGEN, Hilden, Germany) with slight modifications. Each pool was homogenized in 180 µL buffer ATL (QIAGEN) using the TissueLyser II (QIAGEN) with two sets of 96-well adapter plates (QIAGEN) containing 2 mL microcentrifuge tubes (QIAGEN) in which samples were placed, each along with 5 mm diameter stainless steel beads (QIAGEN). The TissueLyser II (QIAGEN) was set to 30 hz/s for a total of 15 min for the pools of whole-mosquito samples and 8 min for the washed cuticle samples, with plates rotated every minute until the end of the homogenization process. Homogenized samples were transferred to new tubes for further processing. Twenty microliters of Proteinase K (QIAGEN) was added to each homogenized sample and incubated at 56 °C overnight, following which 200 µL of buffer AL (QIAGEN) was added and incubated for an additional 2 h at the same temperature. At the end of the incubation period, 200 µL of 100% ethanol was added to the mixture and transferred to spin columns (QIAGEN). The remaining wash steps were performed following QIAGEN’s spin-column protocol for the purification of total DNA from animal tissues, and the purified DNA was eluted in 70 µL buffer AE (QIAGEN). Two negative (sans samples) extraction controls each were processed along with the internal and cuticle surface microbiota samples. All steps (as well as those described below) were performed under sterile conditions. Genomic DNA was stored at −80 °C until further processing.

### PCR for mosquito species identification and 16S rRNA gene amplification

Amplification of the second internal transcribed spacer region (ITS2) of the ribosomal DNA was used to confirm the morphological identification of *An. albimanus*. This was performed on DNA from individual mosquito legs that had been dissected prior to washing the cuticle surface from a subsample of individual adult mosquitoes. The universal ITS2 primers (ITS2 A: TGTGAACTGCAGGACACAT and ITS2 B: TATGCTTAAATTCAGGGGGT) for distinguishing members of cryptic *Anopheles* complexes [27] was used in a PCR reaction volume of 25 µL, comprising ≥100 ng/µL DNA template, 15 µM of each respective primer, 12.5 µL of 2× AccuStart II PCR SuperMix (Quanta Bio, Beverly, MA), and PCR grade water to final volume. Reactions were conducted using a T100™ Thermal Cycler (Bio-Rad, USA) with the following conditions: initial denaturation at 94 °C for 4 min, then 35 cycles of 94 °C for 30 s, 53 °C for 40 s, and 72 °C for 30 s, followed by a final extension for 10 min at 72 °C [28]. Amplified products (~500 bp fragment) resolved by EtBr-stained agarose gel electrophoresis confirmed all samples as *An. albimanus*.

The v3–v4 region of the 16S rRNA gene was amplified from both internal and cuticle surface microbiota samples, along with the negative extraction controls, using the bacterial and archaeal universal primers 341F (**TCGTCGGCAGCGTCAGATGTGTATAAGAGACAG**CCTACGGGNGGCWGCAG) and 805R (**GTCTCGTGGGCTCGGAGATGTGTATAAGAGACAG**GACTACHVGGGTATCTAATCC) with Illumina® (San Diego, CA USA) adapters (in bold). The PCR was performed using a 25 µL reaction volume comprising ≥20 ng/µL DNA template, 5 µM of each primer, 10 µL of 2× KAPA HiFi HotStart PCR mix (Roche, Switzerland), and PCR grade water to final volume. Three negative PCR controls (PCR grade water substituted for DNA template) were processed along with the microbiota samples. Using the aforementioned thermal cycler, the following PCR program was used: initial denaturation at 95 °C for 3 min, then 25 cycles of 95 °C, 55 °C, and 72 °C for 30 s each, followed by a final extension for 5 min at 72 °C. Resulting amplification products (~460 bp) were resolved as described above and quantified using a NanoDrop™ spectrophotometer (Thermo Fisher Scientific, Waltham, MA). These were subsequently purified using Agencourt AMPure XP beads (Beckman Coulter Inc., Indianapolis, IN, USA) at 0.7× (internal) or 0.875× (cuticle surface) sample volume and eluted in 40 µL 10 mM Tris (pH 8.5). Purified PCR products, along with those from all negative controls—which yielded no bands on agarose gel—were submitted to the CDC Biotechnology Core Facility for library preparation and sequencing.

### Library preparation and 16S rRNA sequencing

Index PCR was performed with 25 µL NEBNext High-Fidelity 2X PCR master mix (New England Biolabs Inc., Ipswich, MA), containing 5 µL of each index primer (Nextera XT Index kit v2 set A, B and D; Illumina, San Diego, CA), 10 µL of purified PCR products (0–20 ng/µL) as template, and PCR grade water to final volume of 50 µL. PCR thermal cycler conditions used were: 98 °C for 30 s, followed by 8 cycles of 98 °C for 10 s, 55 °C and 65 °C for 30 s each, followed by a final extension at 65 °C for 5 min. The resulting products were cleaned using Agencourt AMPure XP beads (Beckman Coulter Inc., Indianapolis, IN, USA) at 1.2× sample volume and eluted in 25 µL 10 mM Tris (pH 8.5). Libraries were analyzed for size and concentration, normalized to 2 nM and pooled. For cluster generation, the final 2 nM pool was denatured following Illumina guidelines for loading onto flowcells. Sequencing was performed on an Illumina Hiseq machine, using Hiseq 2 × 250 cycle paired-end sequencing kits. Resulting sequence reads were filtered for read quality, basecalled, and demultiplexed using bcl2fastq (v2.19).

### Sequencing reads’ quality control and filtering

The resulting demultiplexed paired-end sequencing reads (115,250,077 in total, with a maximum length of 250 bp) were imported into the “quantitative insights into microbial ecology” pipeline, QIIME2 v.2017.7.0 [29]. Further read processing and analysis utilizing QIIME2 were performed using v.2018.2.0 of the pipeline. The “divisive amplicon denoising algorithm” DADA2 [30] plugin in QIIME2 was used to “denoise” sequencing reads. This step filters out noise and corrects errors in marginal sequences, removes chimeric sequences and singletons, merges paired-end reads, and finally dereplicates the resulting sequences, resulting in high-resolution amplicon sequence variants (ASVs) for downstream analysis. Using the denoise-paired command, the DADA2 options passed were trunc_len_f: 244, trunc_len_r: 244, and n_reads_learn: 500000, with all other options left as default. This resulted in 30,956,883 ASVs that were further filtered to remove ASVs associated with the extraction and PCR controls (total 4737). In addition, ASVs with a minimum frequency of 100 were removed, resulting in 17,225,776 ASVs ranging from 3277 to 223,222 per sample (Suppl. 2).

### Identification of variables significantly affecting the mosquito microbiota

The variables of interest for this study are presented in Suppl. 3. To identify which covariates affected the microbiota, a multivariate response linear regression model was fit using the q2-gneiss [31] plugin as described in the QIIME2 v.2018.2.0 manual (https://docs.qiime2.org/2018.2/tutorials/gneiss). Gneiss is based on the concept of balances [31], wherein the compositional nature of the data is considered. Inferences on variable-mediated changes in microbial composition are made based on shifts in the balance (i.e., the ratio of abundance) between subsets of the community, rather than absolute or relative abundances of community members, which is currently difficult to infer from microbial composition data. Prior to creating balances, our ASV abundance data were log-transformed, with sample means centered around zero per default q2-gneiss settings. In its simplest form, a balance containing two taxa; one numerator and one denominator $= {\mathrm{log}}\frac{{{\mathrm{abundance}}\;{\mathrm{of}}\;{\mathrm{numerator}}}}{{{\mathrm{abundance}}\;{\mathrm{of}}\;{\mathrm{denominator}}}}$.

In the current study, balances were based on Ward’s hierarchical clustering [32] of ASVs and were calculated using the internal nodes of the resulting tree as described by Morton et al. [31]. The q2-gneiss regression model is fit using balances derived from every subtree from the clustering step. To determine which variables contribute to the shift in balance between community members, the model utilizes a “leave-one-variable-out” approach, where one variable at a time is dropped from the model and the change in R2 is calculated to evaluate the effect of a single covariate on the model. To avoid overfitting the model, q2-gneiss performs a ten-fold cross-validation and reports a mean square error (MSE) for the model and a prediction accuracy MSE at each iteration of the cross-validation process—a lower prediction accuracy MSE indicates the absence of model overfitting. For all fitted models in this study, the prediction accuracy MSE of all ten iterations of the validation process were lower than the model MSE (Suppl 4. and 5.). All the variables outlined in Suppl. 3 were included in the model, individually (Suppl. 4) or as interactions (Suppl. 5.), and any covariate or interaction whose exclusion from the model resulted in an R2 change >0 was scored as affecting the microbiota. A threshold of R2 change ≥0.01 (i.e., explaining 10% of the variation in the microbial composition) was set for significant effect.

### Bacterial diversity

To assess and compare bacterial diversity between samples, the diversity of ASVs within (alpha diversity) and between (beta diversity) samples were computed and compared. The Shannon alpha diversity index (the number of distinct ASVs along with the similarity of their frequencies within a sample) was computed in QIIME2 using the q2-diversity plugin. As alpha diversity metrics are sensitive to uneven sampling depths, multiple rarefactions were performed prior to computing alpha diversity indices, whereby the number of ASVs per sample were randomly selected (without replacement) at an even depth (Suppl. 6). Shannon diversity matrices were compared between samples using the pair-wise Kruskal–Wallis tests with Benjamini–Hochberg false discovery rate (FDR) corrections for multiple comparisons. Bray–Curtis dissimilarity beta diversity index—the compositional dissimilarity in ASV counts between samples—was computed in QIIME2 and visualized by Principal Co-ordinates Analysis (PCoA) plots in R using the phyloseq R package [33]. Also using the q2-diversity plugin in QIIME2, the Bray–Curtis dissimilarity matrices were computed using both even sampling depth (as normalized above), as well as unnormalized ASV counts. For both methods, pair-wise comparisons of the resulting matrices between samples were computed using permutational (999 permutations) multivariate analysis of variance with FDR corrections. There were negligible differences in outputs between the methods, thus visualization in R was conducted using Bray–Curtis dissimilarity matrices on unnormalized ASV counts. Significance for the pair-wise comparisons was set to *q* value (i.e., FDR adjusted *p* value) <0.05.

### Taxonomic annotation and relative abundance of identified bacterial taxa

Taxonomic annotation of ASVs was performed in QIIME 2 using a pre-trained Naive Bayes classifier [34] and the q2-feature-classifier plugin [35]. Prior to annotation, the classifier was trained on the QIIME-compatible 16S SILVA reference (99% identity) database v.128 [36]. The reference sequences were trimmed to span the v3–v4 region (425 bp) of the 16S rRNA gene using the extract-reads command of the q2-feature-classifier plugin. The resulting relative abundance tables of annotated ASVs were exported into R and ggplot2 v.2.2.1 [37] was used to generate stacked bar plots to visualize the relative abundance of bacterial taxa across samples.

### Identification of bacterial taxa affected by pyrethroid exposure

**Fig. 2:**
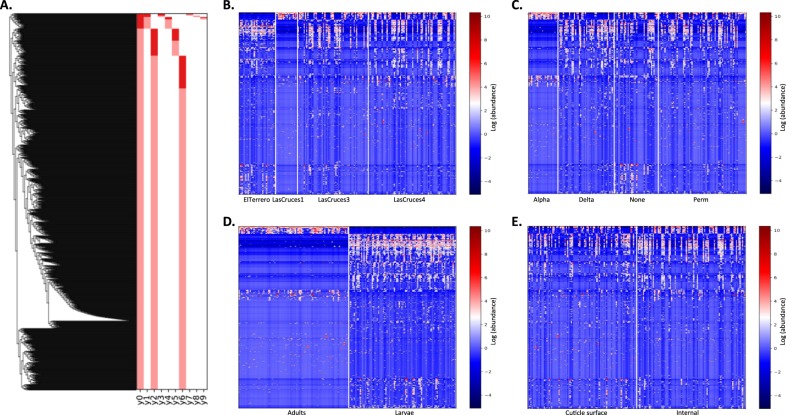
Gneiss linear regression outputs showing variables that contributed to shifts in balance (abundance between subsets) of amplicon sequence variants (ASVs). **a** Hierarchical clustering of ASVs used to create gneiss balances. **b**–**e** are based on this clustering. Red vertical bars indicate the first 10 of 1976 balances (*y*) that were used to build the linear regression model. ASVs that were included as balance numerators are highlighted in light red, while denominators are highlighted in dark red. The first balance, y0, comprised a majority of the ASVs and was the focus of all gneiss analysis. **b**–**e** show heatmaps of ASV abundance across variables that contributed the most to the regression model. The regression model predicted 51% of the shifts in balance between microbial communities, with location (**b**) contributing 9%, insecticide type (**c**) 1%, developmental stage (**d**) 24%, and microbial niche (**e**) 4%. These outputs informed downstream analysis. Alpha alphacypermethrin, Delta deltamethrin, Perm permethrin

Both linear regression analysis and comparison of Bray–Curtis indices indicated that insecticide exposure affected the bacterial composition of *An. albimanus* in this study. Thus, following taxonomic annotation, our goal was to identify ASVs that were differentially abundant between insecticide-exposed and non-exposed mosquitoes. To achieve this, we performed differential abundance analysis using balances in gneiss as described above, since it is currently not possible to infer absolute changes in microbial abundance. Based on the regression analysis outcomes, the internal and cuticle surface microbiota were analyzed separately per insecticide and for each developmental stage. Focusing on the principal balance y0, which comprised a majority of the annotated ASVs (Fig. 2a and Suppl. 4), we compared the log ratios of the components of y0 between exposed and non-exposed samples at both genus and species level. Box plots were used to visualize the distribution of y0 log ratios, and by default, q2-gneiss provides bar plots of the top taxa contributing to shifts in balances.

All data analysis outputs were edited using Inkscape [38]. The raw sequencing reads generated from this project, including those from negative controls, have been deposited in the National Center for Biotechnology Information (NCBI), Sequence Read Archive under the BioProject PRJNA512122.

## Results

### Sample characteristics, 16S rRNA sequencing reads, and quality-control statistics

Suppl. 2 summarizes the number of samples processed for each insecticide per location, the corresponding number of generated sequencing reads, and the number of reads (represented by the percentage of total sequencing reads generated) used for downstream analysis following quality-control and dereplication.

### Variations in bacterial composition are associated with microbial niche and type of pyrethroid exposure

To determine which variables were associated with shifts in *An. albimanus* bacterial composition, we fit a multivariate response linear regression model based on shifts in balance between community members [31] using the entire dataset post quality control and dereplication. The model comprising all variables (Suppl. 3) predicted 51% of the shifts in balance between community members (R2 = 0.51), with insecticide type (R2 = 0.01), microbial niche (R2 = 0.04), location (R2 = 0.09), and developmental stage (R2 = 0.24) having the highest effect on the variation in the bacterial community (Fig. 2b–e). It is worth noting that the variables “Insecticide Type,” “Phenotype,” and “Exposure” are redundant variables because they all contain information about insecticide exposure (Suppl. 3). Thus, when each of the three variables was independently included in the model, the R2 value of each model was 50 ± 1, with insecticide type (R2 = 0.02) and exposure (R2 = 0.02) independently contributing approximately 2% to the variation in bacterial community, while the effect of phenotype remained undetected (Suppl. 7). A total of 1976 balances were created and Suppl. 4 shows the *p* values and R2 calculated for each balance (*y*), with balance y0 comprising a majority of the ASVs (Fig. 2a).

Based on the above outcomes, new models were fit using adult and larval data separately. Only the effect of insecticide type/exposure (R2 = 0.06) and microbial niche (R2 = 0.12) remained in adults (see Suppl. 4 for balance *p* values), with the model explaining 26% of the shifts in balance between adult bacterial community members (R2 = 0.26) (Suppl. 4). In larvae however, location (R2 = 0.18) and microbial niche (R2 = 0.11) were associated with the shifts in balance between community members (see Suppl. 4 for balance *p* values), with 39% of the shifts being explained by the model (R2 = 0.39). This indicates that, following laboratory colonization, the effect of maternal location on microbial composition was reduced or lost in adult F1 progeny of field-collected *An. albimanus* in this study [39]. Fitting another model with the entire dataset based on interactions between variables (Suppl. 5), the effect of microbial niche (R2 = 0.02) remained, this was in addition to the interactions between location and microbial niche (R2 = 0.03) and between insecticide exposure and developmental stage (R2 = 0.01). The model with variable interactions explained 64% of the shifts in balance between overall bacterial communities (Suppl. 5) but did not provide additional insights regarding how the variables affected the bacterial communities. Based on these outcomes, the adult and larval data were individually categorized by microbial niche and used separately for downstream analysis.

### Type of pyrethroid exposure affects bacterial abundance in *An. albimanus*

Comparison of Shannon diversity indices showed a significant effect of both insecticide type and overall exposure on the abundance and evenness of larval cuticle surface bacteria but not internal bacteria, with non-exposed larvae containing the most abundant and even internal and cuticle surface bacterial communities (Suppl. 8). While insecticide exposure significantly affected bacterial abundance on larval cuticle surfaces, there was no significant difference in Shannon diversity indices between pyrethroid-resistant and pyrethroid-susceptible larvae. Conversely, the Shannon diversity index of adult internal bacteria, but not those on the cuticle surface, were significantly affected by type of insecticide (Suppl. 8), with the internal microbiota of deltamethrin-exposed adults representing the most abundant and even bacterial community. No significant effect of overall insecticide exposure was observed on the Shannon diversity index of adult cuticle surface bacteria, and insecticide exposure did not significantly affect the internal bacterial abundance between resistant and susceptible adult mosquitoes (Suppl. 8).

### Insecticide-specific effect of pyrethroid exposure on *An. albimanus* microbiota

Irrespective of the type of pyrethroid exposure, comparisons of Bray–Curtis dissimilarity indices showed significant differences in internal, but not cuticle surface, microbiota between pyrethroid-exposed and non-exposed adults (Suppl. 9). In contrast, there was a significant difference in cuticle surface microbiota between pyrethroid-exposed and non-exposed larvae, which was not evident in the internal microbiota (Suppl. 9). These results were consistent when each type of insecticide tested was considered, except in the case of deltamethrin, where no difference in bacterial composition was detected between exposed and non-exposed mosquitoes (Suppl. 10). In both adults and larvae, the internal and cuticle surface bacteria did not differ between pyrethroid-resistant and pyrethroid-susceptible mosquitoes (Suppl. 9). Also, there were significant differences (*q* < 0.02) in cuticle surface microbiota between alphacypermtherin-exposed and non-exposed adults (Suppl. 10).

Bray–Curtis dissimilarity index comparisons further showed significant differences in bacterial composition between adults and larvae (*F* = 117.4, *p* = 0.001), between internal and cuticle surface microbiota in both larvae (*F* = 39.9, *p* = 0.001) and adult mosquitoes (*F* = 38.7, *p* = 0.001), and between samples exposed to different pyrethroids (Suppl. 11), thus corroborating the results of the linear regression analysis.

**Fig. 3:**
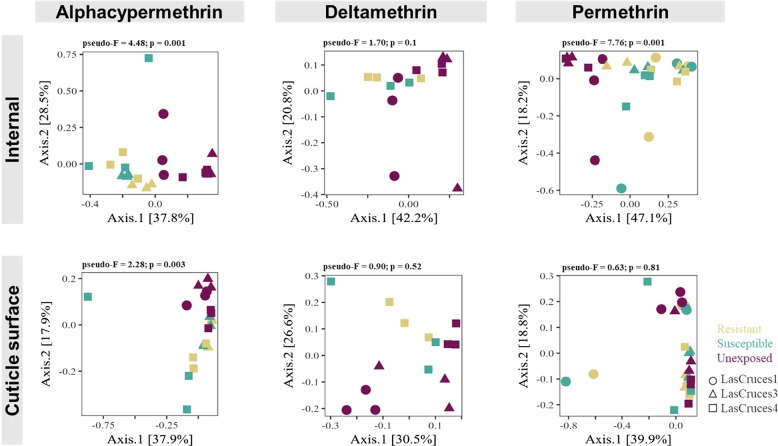
Principal coordinate analysis (PCoA) plots of Bray–Curtis distances between pyrethroid-exposed and non-exposed F_1_ adult *An. albimanus*. PCoA plots, based on Bray–Curtis dissimilarity distances, show clustering patterns of the internal and cuticle surface microbiota in adult F_1_ mosquitoes with respect to pyrethroid exposure. Separate plots are presented for each insecticide tested. Each point on the plots represents the bacterial composition of a pool of three mosquitoes, with the axes representing the first two dimensions of the PCoA, along with the proportion (%) of variation in bacterial composition explained by pyrethroid exposure. Results of overall non-pair-wise beta diversity (Bray–Curtis) comparison using permutational multivariate analysis of variance (999 permutations) tests, presented above each plot, were used to determine whether PCoA patterns were significant. The test statistic value (pseudo-*F*) for each comparison is presented, with significance set to *p* value < 0.05. In general, the plots show distinct separation of pyrethroid-exposed (resistant and susceptible) mosquitoes from non-exposed mosquitoes. These clustering patterns were statistically significant for alphacypermthrin and permethrin but not for deltamethrin in the internal microbiota. The cuticle surface microbiota, however, showed varying clustering patterns with only patterns of alphacypermethrin-tested mosquitoes being consistent with those of the internal microbiota

**Fig. 4:**
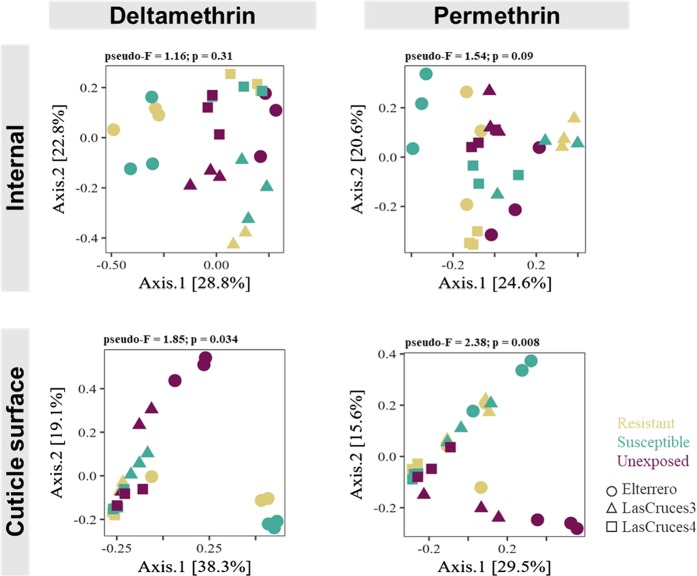
Principal coordinate analysis (PCoA) plots of Bray–Curtis distances between pyrethroid-exposed and non-exposed *An. albimanus* F_1_ larvae. PCoA plots, based on Bray–Curtis dissimilarity distances, show clustering patterns of the internal and cuticle surface microbiota in *An. albimanus* F_1_ larvae with respect to pyrethroid exposure. Separate plots are presented for each insecticide tested. Each point on the plots represents the bacterial composition of a pool of three mosquitoes, with the axes representing the first two dimensions of the PCoA, along with the proportion (%) of variation in bacterial composition explained by pyrethroid exposure. Results of overall non-pair-wise beta diversity (Bray–Curtis) comparison using permutational multivariate analysis of variance (999 permutations) tests, presented above each plot, were used to determine whether PCoA patterns were significant. The test statistic value (pseudo-*F*) for each comparison is presented, with significance set to *p* value < 0.05. For the internal microbiota, the plots show distinct separation of pyrethroid-exposed (resistant and susceptible) mosquitoes from non-exposed mosquitoes, particularly within each location. These clustering patterns, although consistent for both insecticides, were not statistically significant. The cuticle surface microbiota, however, showed a general separation of exposed mosquitoes away from those that were not exposed. This clustering pattern was statistically significant for both insecticides

For each insecticide tested, visualizations of Bray–Curtis diversity distance matrices showed the microbiota of non-exposed mosquitoes clustering distinctly away from those of pyrethroid-exposed mosquitoes. In adults, this clustering pattern was more evident in the internal compared to the cuticle surface microbiota (Fig. 3). However, the reverse was seen in larvae (Fig. 4).

### *Asaia* dominates the internal and cuticle surface microbiota of adult *An. albimanus* but not larvae from the same population

**Fig. 5:**
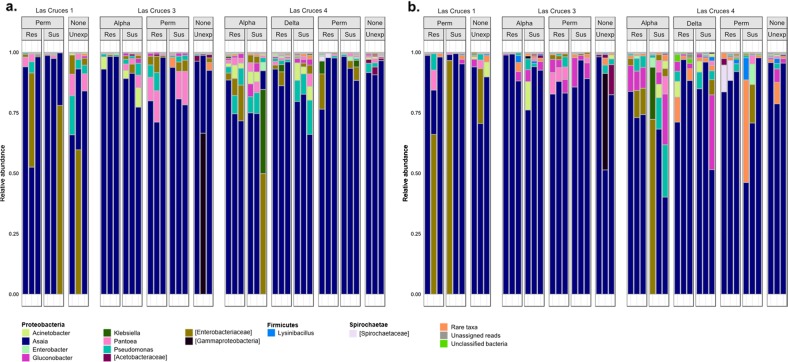
Bar plots showing the relative abundance of taxonomically annotated amplicon sequence variants (ASVs) from F_1_ adult *An. albimanus*. ASVs were taxonomically annotated to the genus level, and only taxa with relative abundance >0.1% are shown, all other taxa are collapsed and presented as “rare taxa.” The bar plots show the relative abundance of annotated ASVs across all sites, sub-categorized by insecticide type and resistance status, indicating that the adult internal (**a**) and cuticle surface (**b**) microbiota is dominated by *Asaia*. Across all insecticides tested, the relative abundance of identified taxa differed between resistant, susceptible, and unexposed mosquitoes in both internal (**a**) and cuticle surface (**b**) microbiota. ASVs that were not identified to the genus level are presented in square brackets, indicating the lowest possible level annotated. Identified taxa are organized by phylum, with phylum name indicated in bold. Res resistant, Sus susceptible, Unexp non-exposed

**Fig. 6:**
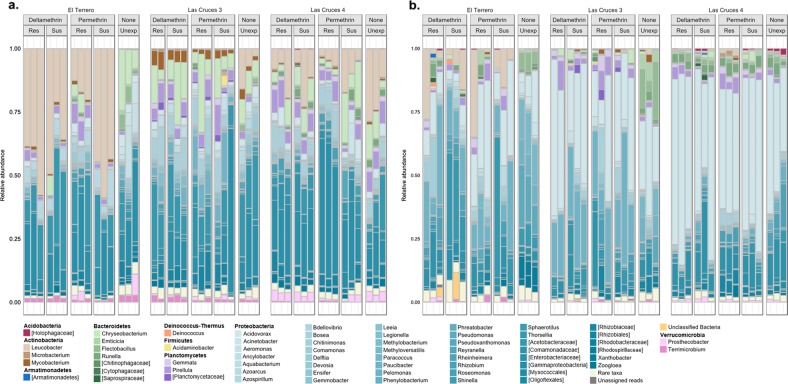
Bar plots showing the relative abundance of taxonomically-annotated amplicon sequence variants (ASVs) from *An. albimanus* F_1_ larvae. ASVs were taxonomically annotated to the genus level, and only taxa with relative abundance >0.1% are shown, all other taxa are collapsed and presented as “rare taxa.” The bar plots, grouped by location and sub-grouped by insecticide type and resistance status, show variable relative abundance of annotated ASVs across all locations, indicating that the larval internal (**a**) and cuticle surface (**b**) microbiota is mostly dominated by bacteria belonging to the phylum *Proteobacteria*. The genus *Leucobacter* was dominant in the internal microbiota, especially in El Terrero and Las Cruces 4 (**a**), while *Acidovorax* dominated the cuticle surface microbiota (**b**). Across all insecticides tested, the relative abundance of identified taxa differed between resistant, susceptible, and non-exposed mosquitoes in both internal (**a**) and cuticle surface (**b**) microbiota. ASVs that were not identified to the genus level are presented in square brackets, indicating the lowest possible level annotated. Identified taxa are grouped and color-themed by phylum, with phylum name indicated in bold. Res resistant, Sus susceptible, Unexp non-exposed

Following taxonomic annotation of ASVs to the genus level, 75 and 140 bacterial genera were detected in adults and larvae, respectively; this was out of a total of 118 (adults) and 203 (larvae) assigned bacterial and archaeal taxa (Suppl. 12 and 13), with archaeal reads only identified in adults. The less diverse adult microbiota predominantly comprised ASVs assigned to the genera *Asaia*, with >70% of these ASVs found in both the internal and cuticle surface microbial niches (Fig. 5). Conversely, ASVs assigned to *Asaia* comprised 0.02% of the overall larval microbiota, with *Leucobacter*, *Thorsellia*, and *Chryseobacterium* dominating the internal microbial niche (collectively constituting 35%), and *Acidovorax* and *Paucibacter* (each making up <50%) dominating the cuticle surface (Fig. 6 and Suppl. 13). Despite being comprised of >70% *Asaia*, the adult cuticle surface microbiota had nearly twice (*n* = 106) as many taxa as the internal microbiota (*n* = 62) (Suppl. 12). Out of the total adult microbial taxa detected (*n* = 118), 47% were unique to the cuticle surface, 10% to the internal microbial niche, and 43% shared by both. In larvae, the cuticle surface microbiota was comprised of only slightly more taxa (*n* = 194) compared to the internal (*n* = 180) microbiota, and out of all taxa detected (*n* = 203), 11% were unique to the cuticle surface, 4% to the internal microbial niche, and 85% were shared by both (Suppl. 13).

### Abundance of *Pseudomonas fragi* and *Pantoea agglomerans* in adult *An. albimanus* are affected by pyrethroid exposure

**Fig. 7:**
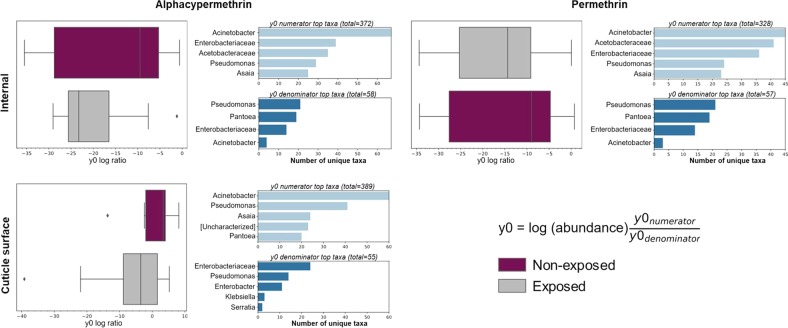
Log ratios of amplicon sequence variants (ASVs) in gneiss balance y0 and the number of unique taxa that contributed to shifts in bacterial composition between insecticide exposed and non-exposed adults. Box plots show the distribution of ASV log ratios between insecticide exposed and non-exposed adult F_1_ progeny. Corresponding horizontal bar plots (right hand side of each panel) show the top unique bacterial taxa in y0 numerator and denominator that contributed to shifts in y0 balance. Separate plots are presented per microbial niche and for each insecticide wherein significant differences in bacterial composition were observed between exposed and non-exposed mosquitoes. The bacterial composition of non-exposed mosquitoes was used as a reference to determine any shifts caused by pyrethroid exposure. Across each insecticide tested, y0 log ratio was lower in insecticide-exposed mosquitoes compared to those that were not exposed, indicating that the abundance of the unique taxa in y0 denominator was higher in insecticide-exposed mosquitoes compared to those that were not and vice versa for the taxa in numerators. This was seen in both internal and cuticle surface microbiota. Taxa were annotated to the genus level where possible or otherwise included in square brackets

**Fig. 8:**
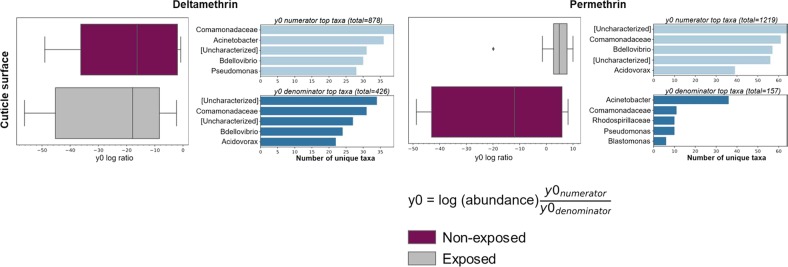
Log ratios of amplicon sequence variants (ASVs) in gneiss balance y0 and the number of unique taxa that contributed to shifts in cuticle surface bacterial composition between insecticide exposed and non-exposed larvae. Box plots show the distribution of ASV log ratios between insecticide-exposed and non-exposed larval F_1_ progeny. Corresponding horizontal bar plots (right hand side of each panel) show the top unique bacterial taxa in y0 numerator and denominator that contributed to shifts in y0 balance. Separate plots are presented for each insecticide wherein significant differences in bacterial composition were observed between exposed and non-exposed mosquitoes. The bacterial composition of non-exposed mosquitoes was used as a reference to determine any shifts caused by pyrethroid exposure. y0 log ratio for deltamethrin-exposed larvae was lower than that of non-exposed larvae, indicating that the abundance of unique taxa in y0 denominator was higher in exposed vs non-exposed larvae. In permethrin-exposed larvae, however, y0 log ratio was higher than those of non-exposed mosquitoes, indicating that the abundance of unique taxa in y0 numerator was higher in exposed compared to non-exposed larvae and vice versa for unique taxa in the denominator. Taxa were annotated to the genus level where possible or otherwise included in square brackets

The relative abundance of bacterial taxa in both larvae (Fig. 6) and adults (Fig. 5) differed between pyrethroid-exposed and non-exposed mosquitoes. In adults, this difference was significant in the internal microbiota for alphacypermethrin and permethrin and in the cuticle surface microbiota for only alphacypermethrin (Fig. 3 and Suppl. 10). In larvae, the difference was only significant in the cuticle surface microbiota for deltamethrin and permethrin (Fig. 4 and Suppl. 10). For each of these significant groups, log ratios of the ASVs making up the principal balance, y0, differed between pyrethroid-exposed and non-exposed groups (Figs. 7 and 8). Since the non-exposed mosquitoes received no treatment, they were used as a reference to determine changes in microbial communities associated with insecticide exposure. In adults, for each insecticide (Fig. 7 and Suppl. 14), the log ratio of balance y0 was lower in pyrethroid-exposed compared to non-exposed mosquitoes in both internal and cuticle surface microbiota, indicating that, in pyrethroid-exposed mosquitoes, the y0 denominators were more abundant than the numerators. At the genus level, unique taxa annotated as *Pseudomonas* and *Acinetobacter* were present in both y0 numerators and denominators of the internal microbiota of alphacypermethrin- and permethrin-exposed adults (Fig. 7), indicating that, within these genera, specific species, strains, and/or isolates were differentially affected by pyrethroid exposure. However, only 20 unique taxa annotated as *Pseudomonas* and <10 assigned to *Acinetobacter* were present in the denominator, compared to >20 and >45 in the numerator, respectively (Fig. 7). Furthermore, in the internal microbiota of both alphacypermethrin- and permethrin-exposed adults, unique taxa annotated as *Pantoea* (*n* = 20 for each insecticide) and *Asaia* (*n* > 20 for each insecticide) were unique to y0 denominator and numerator, respectively (Fig. 7).

Examining balance y0 at the species level revealed that, in the denominator, over half of the taxa annotated as *Pseudomonas* were *P. fragi*, and nearly half of those annotated as *Pantoea* were *P. agglomerans* for each insecticide (Suppl. 14). Overall, ASVs assigned to these bacterial species were more abundant in the internal microbiota of alphacypermethrin- and permethrin-exposed adults (Suppl. 15). For both insecticides, none of the taxa present in the numerator were classified to the species level.

Looking at the cuticle surface composition of alphacypermethrin-exposed adults, unique taxa annotated as *Pseudomonas* were also present in both y0 numerator (*n* = 40) and denominator (*n* = 15) (Fig. 7). However, those annotated as *Asaia* (*n* = 23), *Acinetobacter* (*n* = <60), and *Pantoea* (*n* = 19) were unique to the numerator, indicating that their abundance was lower in the cuticle surface of alphacypermethrin-exposed compared to non-exposed adults. Conversely, those annotated as *Enterobacter* (*n* = 10), *Klebsiella* (*n* = 3), and *Serratia* (*n* = 2) were only present in the denominator. Only *P. fragi* was delineated to the species level (Suppl. 14), and this was only present in the denominator (*n* = 14), indicating that, as in the internal microbiota, it was more abundant in the cuticle surface of alphacypermethrin-exposed compared to non-exposed adults. Overall, this was also true for ASVs classified as *P. fragi* in the cuticle surface of alphacypermethrin-exposed adults (Suppl. 15).

In larval cuticle surface, the log ratio of y0 was lower in deltamethrin-exposed compared to non-exposed mosquitoes, while the opposite was seen for permethrin (Fig. 8). This indicated that the taxa in y0 denominators were more abundant in the cuticle surface of deltamethrin-exposed compared to non-exposed larvae, while the numerators were more abundant in permethrin-exposed compared to non-exposed larvae. On the cuticle surface of deltamethrin-exposed larvae, unique taxa annotated as *Bdellovibrio* were present in both y0 numerator (*n* = 30) and denominator (*n* = 24) (Fig. 8), indicating that, at lower taxonomic levels, members of this genera were differentially abundant between deltamethrin-exposed and non-exposed larvae. However, unique taxa annotated as *Acidovorax* (*n* = 22) were present in y0 denominator, while those annotated as *Acinetobacter* (*n* = 36) and *Pseudomonas* (*n* = 27) were present in the numerator, indicating that the abundance of the former taxa was higher, while that of the latter two taxa was lower, in the cuticle surface of deltamethrin-exposed compared to non-exposed larvae. Overall, this pattern was seen in the abundance of ASVs classified as *Acidovorax*, *Acinetobacter*, and *Pseudomonas* (Suppl. 13). For permethrin, unique bacterial taxa were present in y0 numerator (*Bdellovibrio*, *n* = 58 and *Acidovorax*, *n* = 39) and denominator (*Acinetobacter*, *n* = 38, *Pseudomonas*, *n* = 10, and *Blastomonas*, *n* = 6) (Fig. 8). The larger y0 log ratio of permethrin-exposed compared to non-exposed larvae indicated that the numerators (the same taxa identified as more abundant in deltamethrin-exposed vs non-exposed larvae) were more abundant in the cuticle surface of permethrin-exposed larvae compared to those that were not exposed. The opposite was true for the denominators, but only two of these—*Acinetobacter* and *Pseudomonas—*were also less abundant in the cuticle surface of deltamethrin-exposed larvae. This trend was also evident in the overall abundance of ASVs assigned to these genera (Suppl. 13). None of the bacterial genera whose abundance was affected by insecticide exposure in larvae were classified to the species level (Suppl. 14).

## Discussion

With increasing evidence of microbiota-mediated insecticide resistance in insects, particularly in agricultural pests [12–16], it is plausible that the mosquito microbiota could also contribute to the host’s insecticide detoxification processes. Building upon our earlier findings that showed significant differences in bacterial composition between insecticide-resistant and insecticide-susceptible Peruvian *An. albimanus* [9], we characterized the microbiota of Guatemalan *An. albimanus* with differing pyrethroid insecticide resistance profiles. We focused specifically on late instar larvae (L3–L4) and adult (non-blood-fed virgin females) F_1_ progeny of field-caught mosquitoes to obtain uniform physiological profiles, while maintaining the genetic background of the field populations from where they were collected. Our results showed that insecticide exposure, insecticide type, microbial niche (internal or cuticle surface microbiota), and host developmental stage were significantly associated with shifts in *An. albimanus* microbial composition. To our knowledge, we present the first description of the effects of pyrethroid insecticides on mosquito microbiota and the first description of the microbiota of *An. albimanus* larvae. We also characterized the microbiota on the mosquito cuticle surface for the first time.

Overall bacterial composition differed significantly between pyrethroid-exposed and non-exposed larvae and adults. Considering that the cuticle surface is the first site of insecticide contact, we had anticipated a general effect of insecticide exposure, if present, on the cuticle surface microbiota of both larvae and adults. While this was indeed what we observed in larvae, the result was the opposite for adult mosquitoes, with an effect only detected in the internal microbiota. Our results also showed significant differences in Shannon diversity indices driven by insecticide type and insecticide exposure in adult internal but not cuticle surface microbiota. This insecticide type- and exposure-driven difference in Shannon diversity index was also evident in larvae, but with respect to the cuticle surface microbiota rather than internal microbiota. The differing effect of insecticide type and exposure on larval and adult microbiota could be explained by the differences in how mosquito larvae and adults come into contact with insecticides. Throughout the bioassays, larvae were fully immersed in insecticide-treated water, mimicking their natural exposure to insecticides. Adults were introduced into insecticide-coated bottles (also mimicking natural insecticide exposure) where they mainly contacted the insecticide via their tarsi. As such, the large insecticide-contact area (the entire body surface), as well as their prolonged contact with insecticide, could have caused the change in composition of the cuticle surface microbiota in larvae. Adult malaria vectors typically pick up insecticides on their tarsi upon landing on treated surfaces [40], which may explain the insignificant effect of insecticide exposure on their cuticle surface microbiota.

It is not immediately apparent how pyrethroid insecticide exposure affects the internal microbiota of adult mosquitoes. In insects, pyrethroid insecticides are often metabolized by mixed function oxidase enzymes [41], a characteristic component of internal mosquito tissues including the midgut [42], where they are preferentially produced [43]. The midgut is also where the majority of the internal microbiota is found, so the microbes within this tissue (along with other internal tissues) could conceivably be affected by exposure to pyrethroid insecticides. The internal microbiota of insecticide-exposed adult mosquitoes could also reflect the presence of pre-selected microbiota that is linked to the insecticide resistance status of the host. When individual pyrethroid insecticides were considered, the effects of insecticide exposure were generally consistent across insecticides except for deltamethrin, where no significant effect of exposure was detected on neither the internal nor cuticle surface bacteria in both larvae and adults. Furthermore, in alphacypermethrin-exposed adults, insecticide exposure significantly changed both internal and cuticle surface bacterial composition. The effect of exposure on the cuticle surface microbiota of alphacypermethrin-exposed adults could indicate a more intense resistance to alphacypermethrin in this mosquito population compared to other insecticides. Indeed, bioassays indicated a higher frequency of alphacypermethrin resistance in this mosquito population compared to other insecticides (Suppl. 1C).

Using mosquitoes that were not exposed to insecticides as a reference, we identified unique bacterial taxa in exposed mosquitoes whose abundances changed as a result of pyrethroid exposure. The most probable scenario that could have resulted in this insecticide-induced change in abundance of specific bacterial taxa is that insecticide exposure could have inhibited the growth of certain bacterial taxa and favored the growth of others—especially those that are capable of metabolizing these insecticides, thus resulting in their proliferation. This differential effect of insecticides on bacterial growth has long been demonstrated [44–47], and more recently, insecticides—including permethrin—were shown to alter the microbial community structure in mosquito larval habitats [48]. Here, in both larvae and adults, the abundance of the same bacterial taxa were affected by alphacypermethrin (adults only) and permethrin exposure but not by deltamethrin, which had no significant effect on larval and adult bacterial communities. However, the bacterial taxa affected by pyrethroid treatments were different between larvae and adults.

In larval cuticle surface microbiota, the abundance of unique taxa annotated as *Bdellovibrio* and *Acidovorax* was higher in insecticide-exposed compared to non-exposed mosquitoes, while that of taxa annotated as *Acinetobacter*, *Pseudomonas*, *Blastomonas*, and also *Bdellovibrio* was lower in insecticide-exposed mosquitoes for both deltamethrin and alphacypermethrin. The identification of differential abundance of unique *Bdellovibrio* taxa in insecticide-exposed larvae suggests that pyrethroid exposure had differential effects on this group of bacteria, and potentially others, at lower taxonomic levels. These differentially abundant bacterial taxa in pyrethroid-exposed larvae were not classified beyond the genus level. Nonetheless, bacteria belonging to the genera *Bdellovibrio* have been shown to decrease in abundance upon exposure to the insecticide malathion [49] but how they are affected by pyrethroid insecticide has not yet been described. Likewise, information is lacking on the effect of pyrethoids on *Acidovorax*. In support of the notion that pyrethroid exposure could have differential effects on highlighted bacterial genera at lower taxonomic levels, several strains of *Acinetobacter* [50–52], as well as *Pseudomonas* [53–55]—both genera whose abundance was lower in pyrethroid-exposed larvae in the current study—have been shown to metabolize pyrethroid insecticides, including deltamethrin and permethrin. It is unclear how pyrethroids affect bacteria belonging to the genus *Blastomonas*, as this genus has been proposed for reclassification (without agreement by several authorities) as *Sphingomonas* [56–58], a known pyrethroid-degrading genus [59–62].

Unique taxa annotated as *P. fragi* and *P. agglomerans* were more abundant in the internal microbiota of both alphacypermethrin- and permethrin-exposed adults. Likewise, in adult cuticle surface microbiota, the abundance of unique taxa annotated as *P. fragi* was higher in alphacypermethrin-exposed adults compared to those that were not. While unique taxa annotated as *Acinetobacter*, *Pseudomonas*, and *Asaia* were more abundant in the internal and cuticle surface microbiota of non-exposed compared to alphacypermethrin- and permethrin-exposed adults, they were not classified beyond the genus level. Although unique taxa annotated as *P. agglomerans* were more abundant in the internal microbiota of both alphacypermethrin- and permethrin-exposed adults, those annotated as *Pantoea*, but not to the genus level, were more abundant in the cuticle surface of non-exposed adults, further demonstrating that insecticide exposure had differential effects on the same bacterial genera at lower taxonomic levels. In addition, the abundance of taxa annotated as *Klebsiella* and *Serratia*—both known to metabolize pyrethroids [8, 63–65]—was higher in the cuticle surface of alphacypermethrin-exposed compared to non-exposed adults, but they were not classified to the species level and their overall abundance in adult cuticle surface was low. Several strains of *Pseudomonas*, particularly those of *P. fluorescence* have been shown to metabolize pyrethroid insecticides [54, 55, 66], including permethrin. However, little is known of the effects of pyrethroids on *P. fragi*. Similarly, there is scarce information about the effects of pyrethroids on *P. agglomerans* and *Pantoea* in general. However, *Pantoea* has been identified in insecticide-resistant insects including *An. albimanus* [9, 67]. Insecticide-degrading enzymes in *P. agglomerans* isolated from diamondback moths have also been described [68, 69], demonstrating their role in insecticide metabolism within insects.

Insect internal microbiota have been shown to contribute to the metabolism of topically applied insecticides, consequently contributing to host insecticide resistance [12]. Our results show a significant effect of pyrethroid exposure on the bacterial composition of *An. albimanus* microbiota, and we have also identified insecticide-metabolizing bacterial taxa in insecticide-exposed mosquitoes. The results presented here, using adult F_1_ progeny from field-collected mosquitoes, along with those from our previous work on field-collected adult mosquitoes [9], suggest that insecticide exposure could be selecting for insecticide-metabolizing bacteria. As has been demonstrated in other insects [12, 68–70], these bacteria could also be contributing to insecticide metabolism within mosquitoes, consequently augmenting the host’s ability to tolerate insecticide exposure.

Our previous findings showed significant differences in bacterial composition between mosquitoes that were susceptible to the diagnostic dose of fenitrothion and those that were resistant to five times the diagnostic dose [9]. However, in the present study, we only considered mosquitoes that were susceptible or resistant to the diagnostic dose of the insecticides due to the low intensity of insecticide resistance in the study area. This low insecticide resistance intensity could explain why the bacterial composition did not differ significantly between insecticide-resistant and insecticide-susceptible mosquitoes in this case. Regardless, exposure to insecticides significantly affected bacterial composition, indicating that ongoing exposure to insecticides in the environment could continue to select for insecticide-metabolizing bacteria. This may be particularly important with respect to insecticide resistance intensity. The underlying mechanisms of insecticide resistance intensity in mosquitoes are poorly understood, and we propose that the metabolism of insecticides by host microbiota could be a contributing factor.

As has previously been shown [71], larval microbiota was more diverse than adult microbiota in this study, and overall, the identified bacterial taxa have previously been identified in *Anopheles* mosquitoes [11, 72], including Latin American *Anopheles* [9, 72]. While we characterized the bacterial composition of *An. albimanus* larvae for the first time in this study, our results included bacterial taxa that have been identified in other *Anopheles* larvae [71, 73]. Unlike in humans where the skin surface microbiota is well characterized, studies on insect cuticle surface is sparse. Human skin surface is comprised of less diverse bacterial communities compared to the internal organs (collectively) [74]. Similarly, a survey of the cuticle surface microbiota of Canadian dark beetles revealed a more diverse internal microbiota compared to the cuticle surface [75]. However, in this study, we report that in both mosquito larvae and adults, the cuticle surface contains more diverse bacteria than the internal microbial niche, which may be because of a simpler diet in mosquitoes compared to the aforementioned organisms. As has previously been reported [76], lower diversity of internal microbiota could also indicate a conserved internal bacterial community in mosquitoes, thus corroborating earlier reports of the presence of an internally selective environment [71]. While our data showed that the mosquito cuticle surface harbors a more diverse bacterial community compared to the internal microbial niche, 85% and 43% of the bacterial taxa were shared by both internal and cuticle surface microbiota in larvae and adults, respectively. This suggests that bacteria on the cuticle surface are not only incidentally acquired from the host’s environment. The relationship between bacterial composition of the mosquito cuticle surface and the host environment remains poorly understood, and it remains unknown how the cuticle surface microbiota may contribute to the host’s physiological processes.

We also report the detection of *Asaia* in *An. albimanus* for the first time and showed that it dominated (>70%) the adult internal and cuticle surface microbiota. While laboratory-reared *Anopheles* have been shown to predominantly harbor *Asaia* [77], studies identifying *Asaia* in field-collected *Anopheles* have commonly been qualitative [78, 79], with sparse documentation of variable *Asaia* abundance. *Asaia* was also present in larval microbiota, albeit in negligible proportions (0.02%) (noting that the larvae tested were from the same parents and generation as the adults tested). Thus supporting the notion that, while the microbiota may be transient in immature mosquito stages due to multiple molting events, rapid development, and physiological changes [80], some bacteria can be transstadially transmitted to the adult stage [6]. Furthermore, the predominance of *Asaia* in adults despite their negligible proportions in larvae is strongly indicative of *Asaia*’s ability to quickly and efficiently colonize and dominate the microbiota of adult *Anopheles* mosquitoes [77], thus resulting in its consideration as a paratransgenesis candidate for malaria control [81]. Moreover, since we used non-blood-fed virgin adult females in this study, the overabundance of *Asaia* in adults (which may also have been acquired independently of the larval stage) could be an indicator of the host’s age and/or feeding status. This is because mosquito age and feeding status are known to affect bacterial composition [82], and *Asaia* is a known component of sugar sources [83]. Our sequencing approach deeply sampled the mosquito microbiota, as indicated by the plateaued rarefaction curves (Suppl. 6), thus identifying bacterial taxa in each sample beyond the point at which further sequencing had no effect on the abundance of detected bacterial communities. The disparity detected in abundance of *Asaia* between larvae and adults is thus biologically relevant and not a sequencing artifact. While a few studies have shown that the mosquito microbiota varies across host’s developmental stages [71, 84], more work is still needed to understand when and how the mosquito microbiota changes throughout host development and how these changes might affect host physiology with regard to insecticide resistance and other key characteristics, such as vector competence.

The results presented here highlight differential effects of insecticide exposure on the mosquito microbiota across mosquito developmental stages and insecticide types and indicate the presence of a conserved microbiota—particularly within adult mosquitoes—that is altered by insecticide exposure. Insecticide-metabolizing bacterial taxa were also identified in pyrethroid-exposed *An. albimanus*. Together, these findings demonstrate how insecticide pressure in the environment could be altering mosquito bacterial composition by favoring insecticide-metabolizing communities and thus potentially contributing to insecticide resistance. Focusing on the bacterial taxa that were more abundant in pyrethroid-exposed mosquitoes, future work will characterize specific bacterial components that are being affected by pyrethroid exposure and quantify their contribution to resistance in mosquitoes.

## Supplementary information

Suppl. 1

Suppl. 2

Suppl. 3

Suppl. 4

Suppl. 5

Suppl. 6

Suppl. 7

Suppl. 8

Suppl. 9

Suppl. 10

Suppl. 11

Suppl. 12

Suppl. 13

Suppl. 14

Suppl. 15
