# Supplementary material for: Pyrethroid exposure alters internal and cuticle surface bacterial communities in Anopheles albimanus
Source: ISME J. 2019 Jun 6;13(10):2447–64. doi: 10.1038/s41396-019-0445-5 (PMC6776023; doi:10.1038/s41396-019-0445-5)
Supplement: Supplementary file 2 — Suppl. 2 [file 41396_2019_445_MOESM2_ESM.pdf]

Suppl. 2: Sequencing outputs and proportion of reads used for downstream analysis following quality control and dereplication.

			Number of reads (% remaining following QC, dereplication and chimera removal)					
			Internal microbiota			Cuticle surface microbiota		
**Stage**	**Location**	**Insecticide**	**Resistant**	**Susceptible**	**Unexposed**	**Resistant**	**Susceptible**	**Unexposed**
**Adults**	Las Cruces 1	Permethrin	2495656 (60)	2421158 (59)	2356223 (61)	1847318 (64)	1973328 (58)	1220490 (75)
	Las Cruces 3	Alphacypermethrin	2575153 (75)	2279853 (66)	2697153 (71)	1451582 (85)	1602746 (80)	1687593 (85)
		Permethrin	2434964 (62)	2035362 (62)		1685611 (85)	1723938 (89)	
	Las Cruces 4	Alphacypermethrin	2403001 (65)	2224020 (48)	1996979 (79)	1548342 (81)	1905025 (45)	1379612 (91)
		Deltamethrin	1657741 (74)	1999803 (66)		1018949 (85)	1208000 (82)	
		Permethrin	1552101 (62)	1030681 (69)		1437977 (87)	1350102 (79)	
**Total**			**13118616 (66)**	**11990877 (61)**	**7050355 (70)**	**8989779 (80)**	**9763139 (70)**	**4287695 (84)**
**Larvae**	El Terrero	Deltamethrin	2285602 (36)	2417346 (34)	3160044 (26)	807512 (46)	174347 (34)	2042499 (35)
		Permethrin	2440043 (31)	2468291 (35)		1732541 (50)	1054643 (47)	
	Las Cruces 3	Deltamethrin[a]	1187935 (35)	2698429 (30)	2983923 (30)	1203630 (57)	1671137 (48)	2740549 (44)
		Permethrin	2266161 (33)	2651416 (32)		1656194 (45)	1629694 (47)	
	Las Cruces 4	Deltamethrin	1387104 (35)	3239030 (25)	2768185 (30)	2005520 (66)	1589326 (60)	1505523 (55)
		Permethrin	2120366 (33)	2636276 (33)		1760717 (57)	1731822 (61)	
**Total**			**11687211 (34)**	**16110788 (31)**	**8912152 (28)**	**9166114 (54)**	**7850969 (53)**	**6288571 (44)**

[a] Only two pools of larvae were processed for the deltamethrin resistant category

We characterized the internal and cuticle surface microbiota of whole larvae (n=132) and adult (135) F_1 progeny of wild caught *Anopheles albimanus* from four locations in La Gomera, Guatemala using 16S rRNA gene sequencing. These mosquitoes had been categorized as resistant, susceptible or unexposed to pyrethroid insecticides using the CDC bioassay, and for sequencing, were consolidated into 44 and 45 pools of larvae and adults respectively. Each pool comprised 3 mosquitoes, and 3 pools were processed per category (i.e. resistant, susceptible or unexposed). A total of 55,200,461 (adults) and 60,015,805 (larvae) sequencing reads were obtained and processed for downstream analysis. The table provides a breakdown of sequencing reads per category and the proportion of reads used for downstream analysis.
