# Supplementary material for: Pyrethroid exposure alters internal and cuticle surface bacterial communities in Anopheles albimanus
Source: ISME J. 2019 Jun 6;13(10):2447–64. doi: 10.1038/s41396-019-0445-5 (PMC6776023; doi:10.1038/s41396-019-0445-5)
Supplement: Supplementary file 3 — Suppl. 3 [file 41396_2019_445_MOESM3_ESM.pdf]

Suppl. 3: variables included in regression model

S/N	Variables	Description	Categories
1	Location	Collection site of parent population	Las Cruces 1, Las Cruces 3, Las Cruces 4 or El Terrero
2	Phenotype	Pyrethroid resistance phenotype based on outcomes of	Resistant, Susceptible, or Unexposed
3	Type of insecticide	Type of pyrethroid insecticide tested	permethrin, alphacypermethrin, deltamethrin or none
4	Insecticide exposure		Exposed (i.e. Resistant & Susceptible) or unexposed
5	Developmental stage		Larva (L3-L4) or adult (2-5 d)
6	Microbial Niche	Collection site of the microbiota	Cuticle surface or internal
