# Supplementary material for: Pyrethroid exposure alters internal and cuticle surface bacterial communities in Anopheles albimanus
Source: ISME J. 2019 Jun 6;13(10):2447–64. doi: 10.1038/s41396-019-0445-5 (PMC6776023; doi:10.1038/s41396-019-0445-5)
Supplement: Supplementary file 6 — Suppl. 6 [file 41396_2019_445_MOESM6_ESM.pdf]

Suppl. 6: Rarefaction curves & depth

The following figures show rarefaction curves of Shannon alpha diversity index for each mosquito pool (3 mosquitoes/pool) processed by developmental stage, microbial niche and type of insecticide tested. All curves plateaued, indicating that additional sampling efforts did not result in changes in abundance and evenness of microbial taxa per sample. At each sampling depth shown, each curve shows the average Shannon diversity value, along with the range (boxplots—minimum, median and maximum) of values from 10 rarefaction iterations

Alphacypermethrin: adult cuticle surface

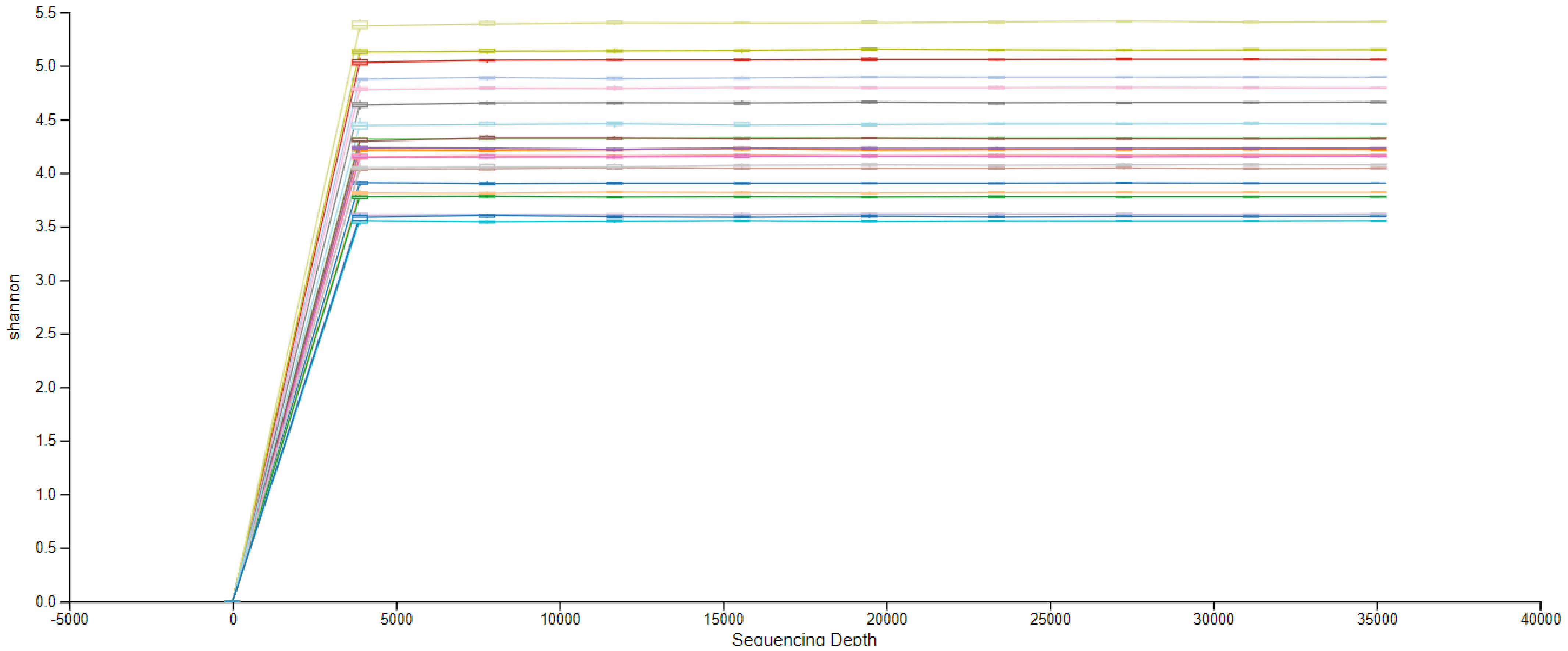

Alphacypermethrin: adult internal microbiota

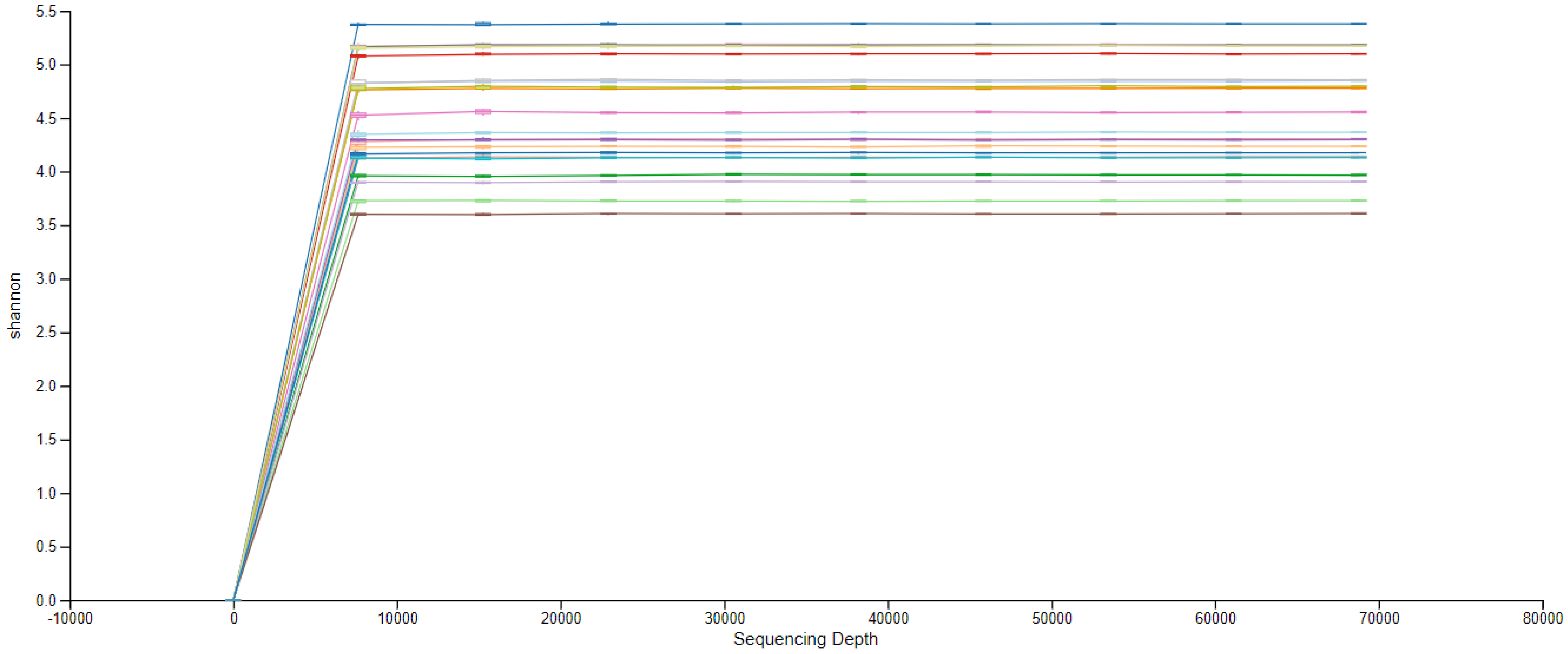

Deltamethrin: adult cuticle surface

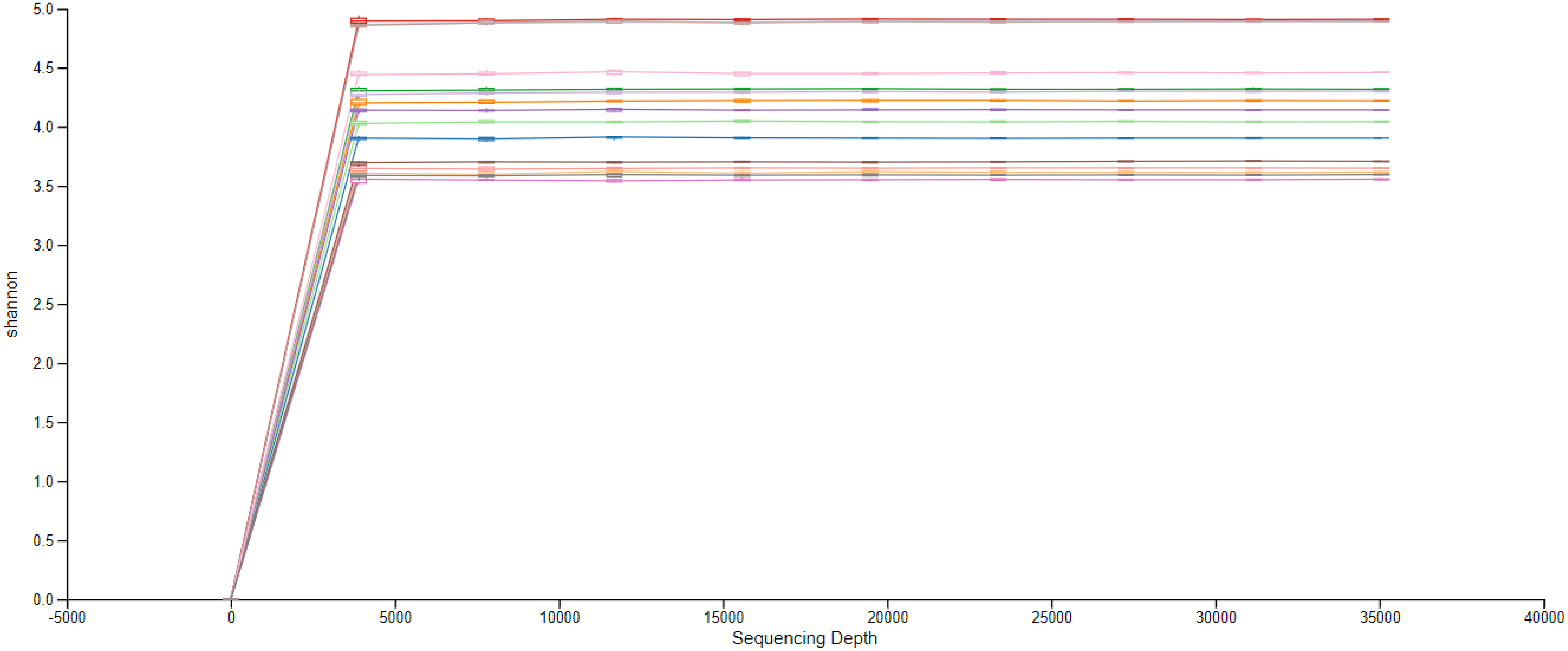

Deltamethrin: adult internal microbiota

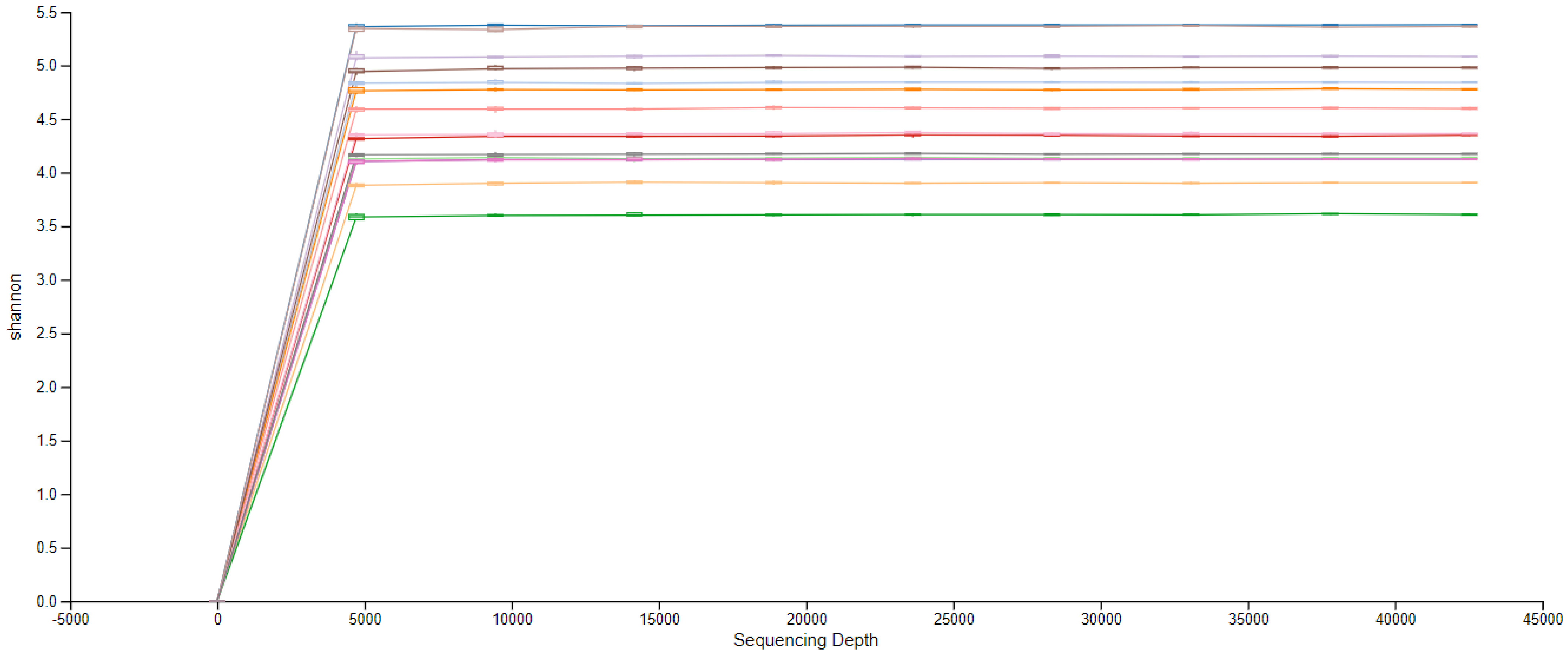

Permethrin: adult cuticle surface

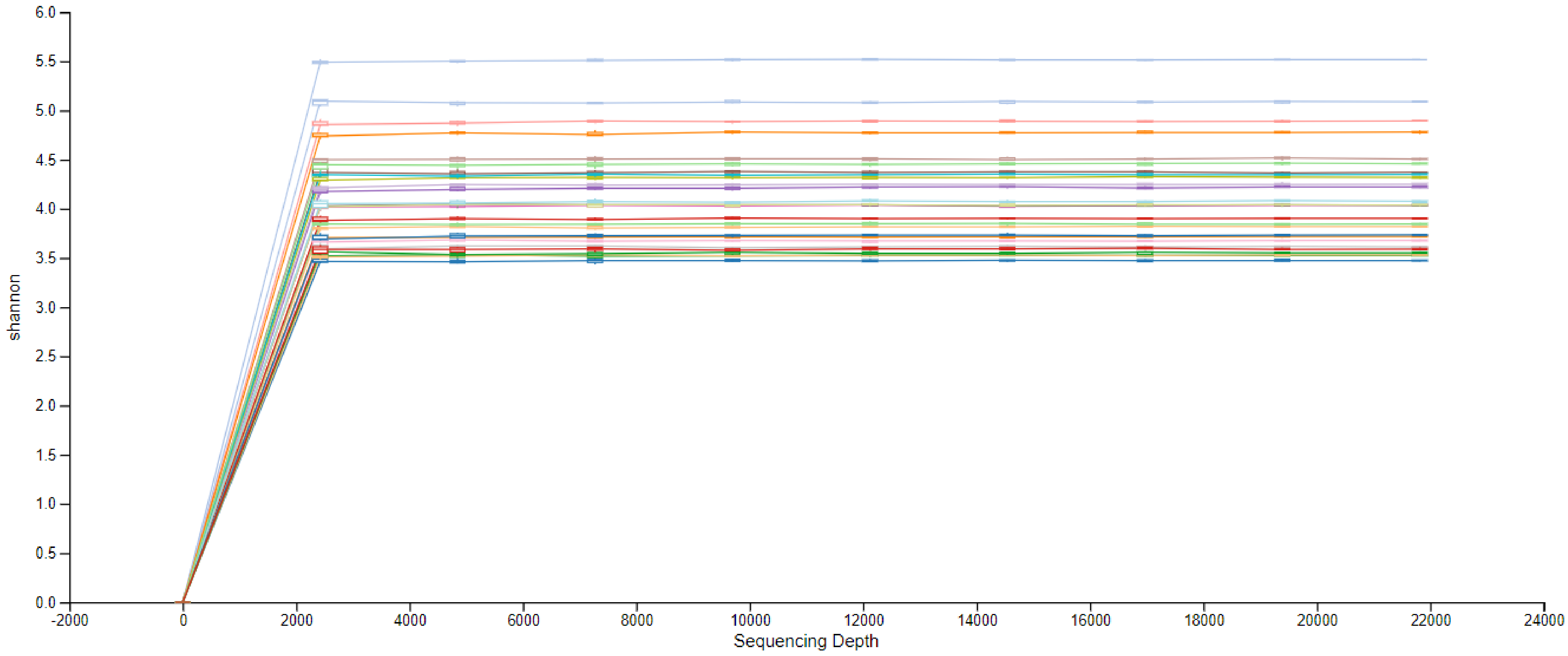

Permethrin: adult internal microbiota

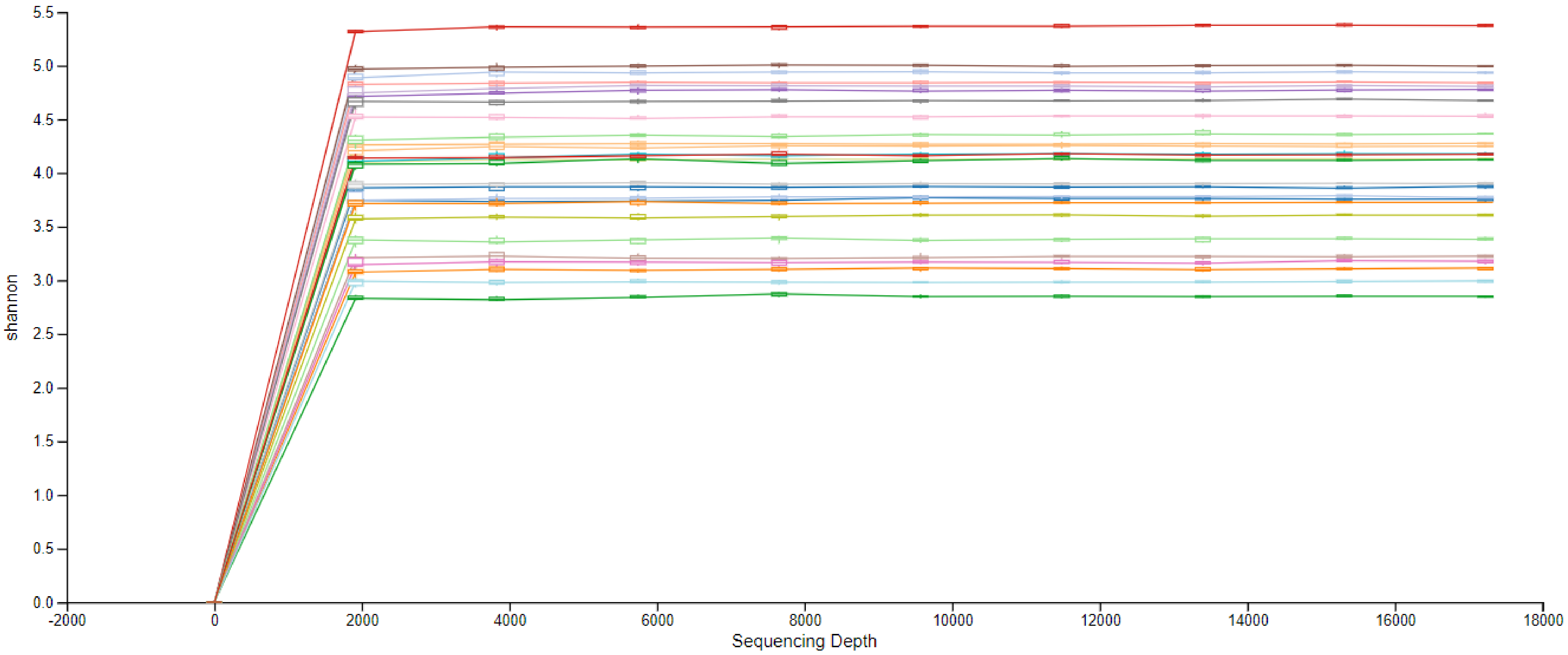

Deltamethrin: larval cuticle surface

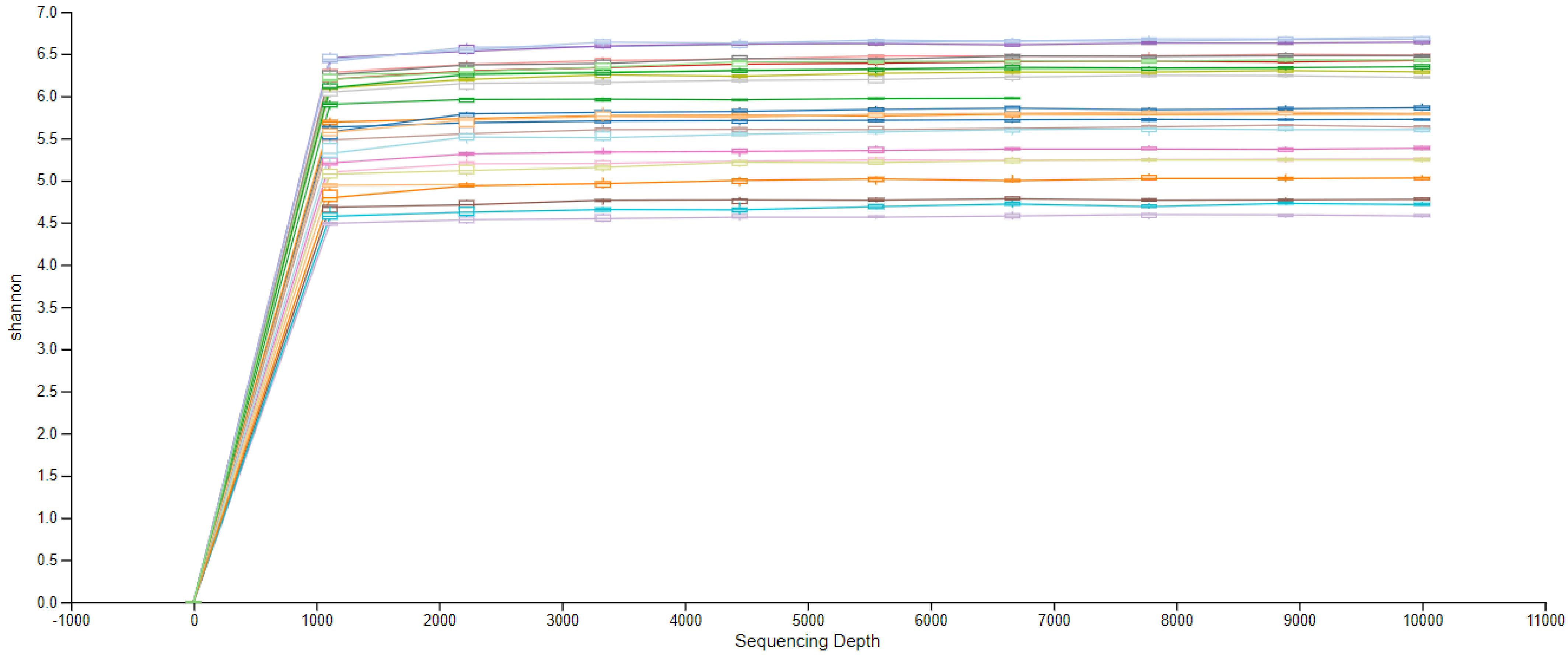

Deltamethrin: larval internal microbiota

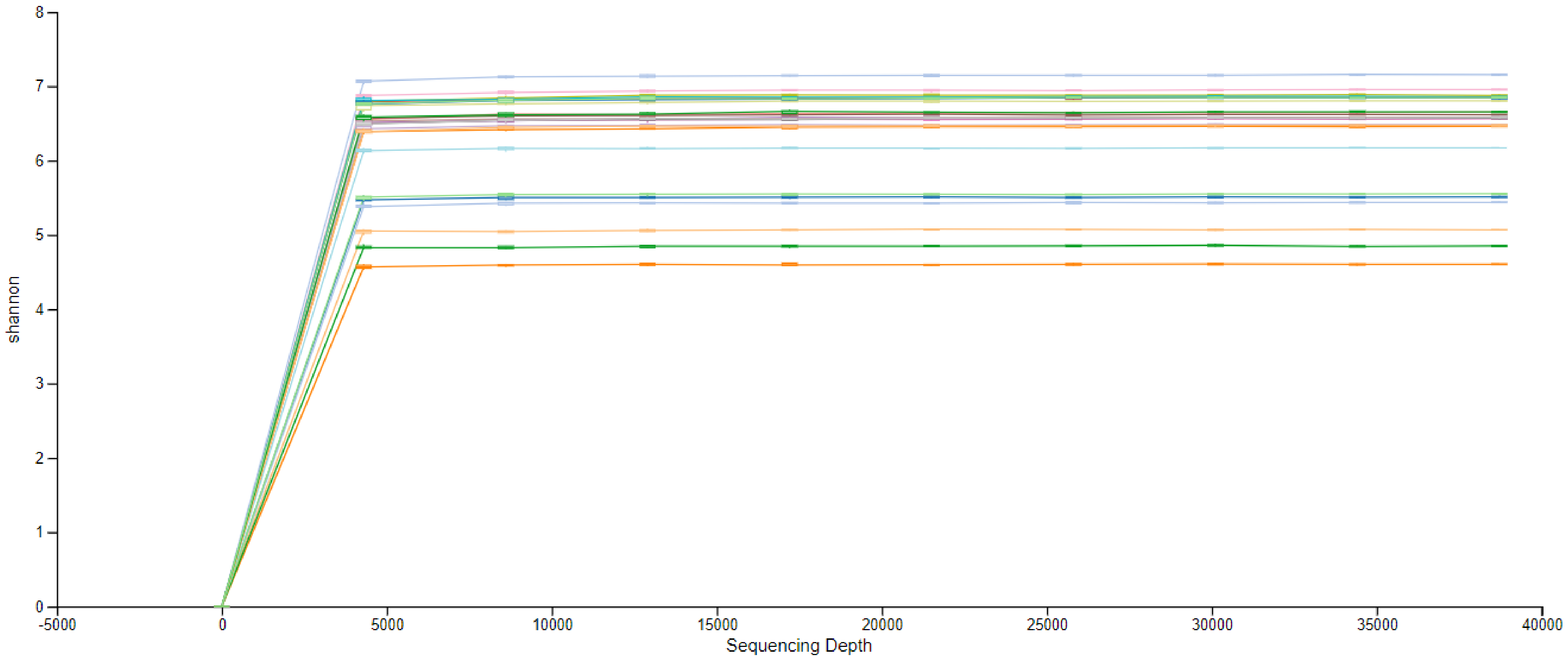

Permethrin: larval cuticle surface

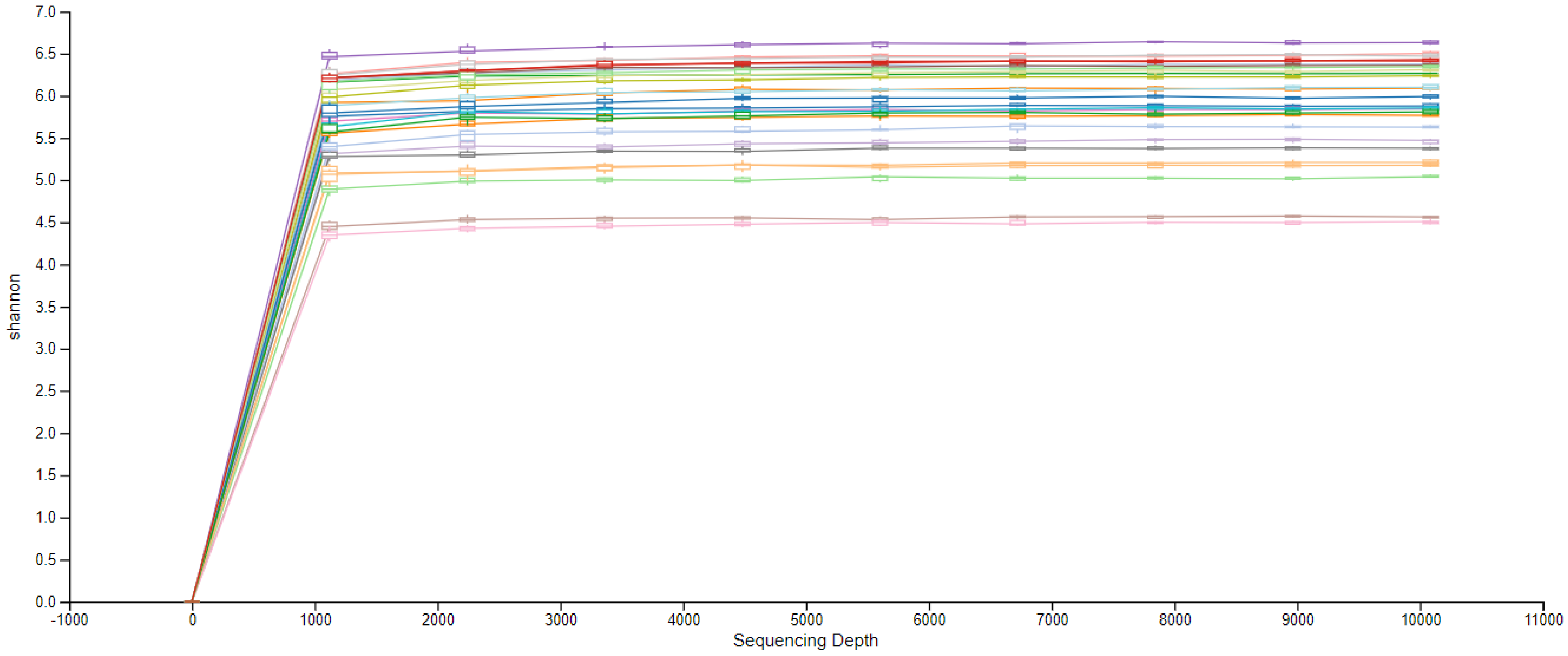

Permethrin: larval internal microbiota

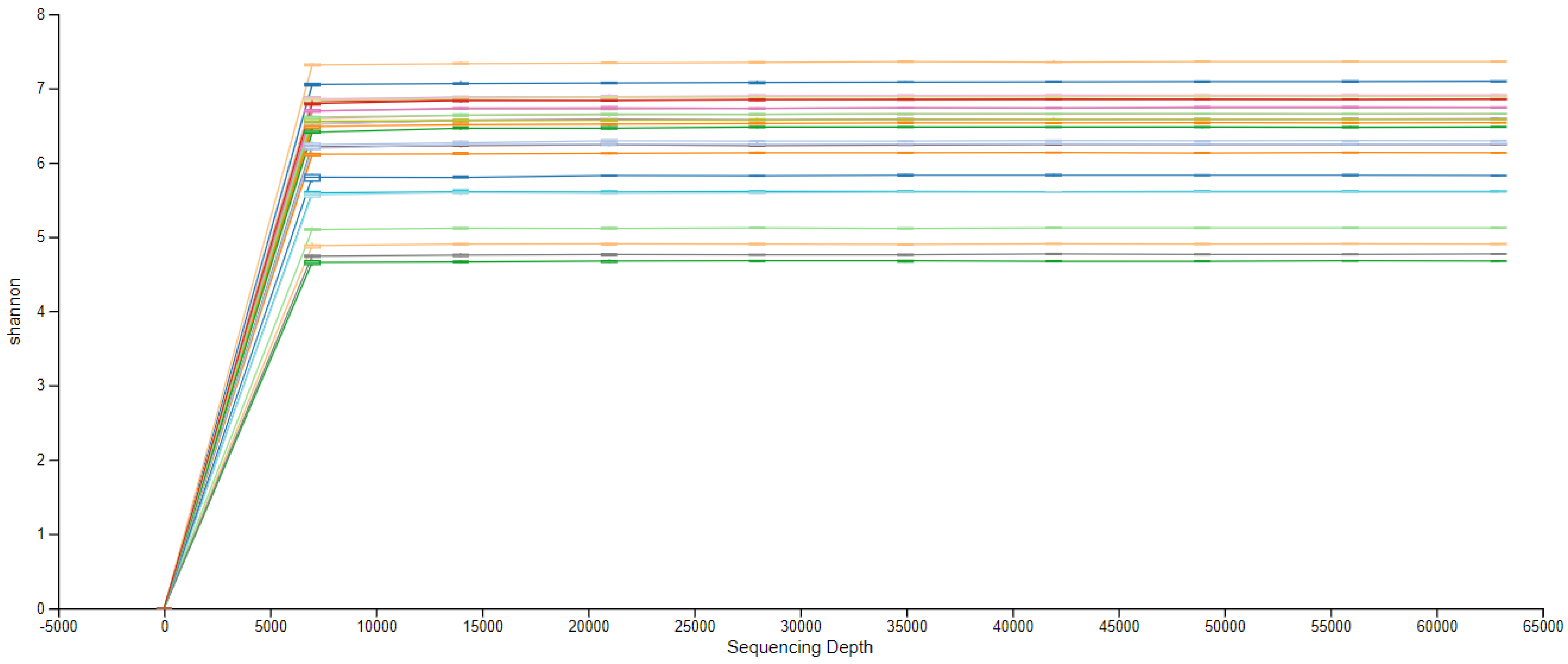

Depth of rarefaction across categories.

Sequencing reads ranged from 3,277 - 223,222 across samples, and to obtain an even sampling depth for alpha diversity analysis, samples were rarefied to at least 10000 reads/sample across categories - a depth that was sufficient to capture the overall composition of each sample. In categories with more reads, rarefaction was set to the lowest number of reads in that category.

		Rarefaction depth	
Stage	Insecticide	Internal microbes	Microbes on surface of the cuticle
Adults	Alphacypermethrin	68781	35066
	Deltamethrin	42524	35066
	Permethrin	17227	21817
Larvae	Deltamethrin	38715	10000*
	Permethrin	62928	10086

*lowest number of reads in this categpry was 3277. Three pools of delatamethrin susceptible samples from El Terrero were excluded from diversity analysis at this depth
