# Supplementary material for: Pyrethroid exposure alters internal and cuticle surface bacterial communities in Anopheles albimanus
Source: ISME J. 2019 Jun 6;13(10):2447–64. doi: 10.1038/s41396-019-0445-5 (PMC6776023; doi:10.1038/s41396-019-0445-5)
Supplement: Supplementary file 9 — Suppl. 9 [file 41396_2019_445_MOESM9_ESM.pdf]

Suppl. 9: Beta diversity comparisons show differential bacterial composition between pyrethroid-exposed and non-exposed *An. albimanus*

			Internal microbiota				Cuticle surface microbiota			
Stage	Group 1	Group 2	N	pseudo-F	p-value	q-value	N	pseudo-F	p-value	q-value
Adults	Resistant	Susceptible	36	0.46	0.843	0.843	36	1.10	0.340	0.340
	Resistant	Non-exposed	27	**11.80**	**0.001**	**0.002**	27	1.71	0.102	0.306
	Susceptible	Non-exposed	27	**9.44**	**0.001**	**0.002**	27	1.27	0.240	0.340
Larvae	Resistant	Susceptible	35	1.06	0.335	0.335	32	1.04	0.340	0.340
	Resistant	Non-exposed	26	1.91	0.072	0.216	26	**3.28**	**0.005**	**0.008**
	Susceptible	Non-exposed	27	1.49	0.182	0.273	24	**3.92**	**0.004**	**0.008**

Pair-wise comparisons of beta diversity (Bray Curtis) between pyrethroid-exposed and non-exposed mosquitoes showed significant differences in adult internal, but not cuticle surface, bacterial composition. In contrast, this difference was evident in larval cuticle surface, but not internal, microbiota. There was no significant difference in bacterial composition between pyrethroid-resistant and –susceptible mosquitoes at both larval and adult stages. Comparisons were conducted using PERMANOVA (999 permutations) tests with Benjamini-Hochberg FDR correction (q-value). Significance is set to q-value (adjusted p-value) <0.05. N = number of mosquito pools included in the analysis; each pool comprised 3 mosquitoes.
