# Supplementary material for: Pyrethroid exposure alters internal and cuticle surface bacterial communities in Anopheles albimanus
Source: ISME J. 2019 Jun 6;13(10):2447–64. doi: 10.1038/s41396-019-0445-5 (PMC6776023; doi:10.1038/s41396-019-0445-5)
Supplement: Supplementary file 10 — Suppl. 10 [file 41396_2019_445_MOESM10_ESM.pdf]

Suppl. 10: Beta diversity comparisons show differential bacterial composition between *Anopheles albimanus* that exposed or unexposed to alphacypermethrin and permethrin.

				Internal microbiota				Cuticle surface microbiota			
Stage	Insecticide	Group 1	Group 2	N	pseudo-F	p-value	q-value	N	pseudo-F	p-value	q-value
Adults	Alphacypermetrin	Resistant	Susceptible	12	1.48	0.128	0.128	12	1.39	0.134	0.134
		Resistant	Unexposed	15	**6.05**	**0.001**	**0.002**	15	**3.10**	**0.014**	**0.021**
		Susceptible	Unexposed	15	**5.16**	**0.001**	**0.002**	15	**2.59**	**0.008**	**0.021**
	Deltamethrin	Resistant	Susceptible	6	1.01	0.421	0.421	6	0.53	1.000	1.000
		Resistant	Unexposed	12	1.49	0.173	0.260	12	1.23	0.318	0.843
		Susceptible	Unexposed	12	2.17	0.083	0.249	12	0.81	0.562	0.843
	Permethrin	Resistant	Susceptible	17	0.37	0.789	0.789	18	0.45	0.684	0.770
		Resistant	Unexposed	18	**12.29**	**0.001**	**0.002**	18	0.85	0.595	0.770
		Susceptible	Unexposed	17	**9.64**	**0.001**	**0.002**	18	0.66	0.770	0.770
Larvae	Deltamethrin	Resistant	Susceptible	17	0.41	0.810	0.810	16	0.76	0.538	0.538
		Resistant	Unexposed	17	1.55	0.130	0.245	17	2.38	0.051	0.077
		Susceptible	Unexposed	18	1.54	0.163	0.245	17	2.35	0.043	0.077
	Permethrin	Resistant	Susceptible	18	1.47	0.180	0.270	17	1.11	0.315	0.315
		Resistant	Unexposed	18	1.99	0.053	0.159	18	**2.90**	**0.018**	**0.030**
		Susceptible	Unexposed	18	1.22	0.280	0.280	17	**2.95**	**0.020**	**0.030**

Pair-wise comparisons of beta diversity (Bray Curtis) between insecticide-exposed (i.e. resistant and susceptible) and –unexposed mosquitoes show variable results when individual insecticides were considered. In adults, the bacterial composition of mosquitoes exposed (i.e. resistant and susceptible) to alphacypermethrin and permethrin differed significantly from those of unexposed mosquitoes. For both insecticides, this difference was evident in the internal microbiota, but only true for alphacypermethrin in the cuticle surface microbiota. Conversely, the bacterial composition on larval cuticle surface, but not the internal microbial niche, differed only between permethrin exposed and unexposed mosquitoes. No effect on bacterial composition was seen in deltamethrin-tested larvae or adults. Also, for each insecticide tested, in both larvae and adults, there was no significant difference in bacterial composition between insecticide resistant or susceptible mosquitoes. Comparisons were conducted using PERMANOVA (999 permutations) tests with Benjamini-Hochberg FDR correction (q-value). Significance is set to q-value (adjusted p-value) <0.05. N = number of mosquito pool included in the analysis; each pool comprised 3 mosquitoes.
