# Supplementary material for: Pyrethroid exposure alters internal and cuticle surface bacterial communities in Anopheles albimanus
Source: ISME J. 2019 Jun 6;13(10):2447–64. doi: 10.1038/s41396-019-0445-5 (PMC6776023; doi:10.1038/s41396-019-0445-5)
Supplement: Supplementary file 11 — Suppl. 11 [file 41396_2019_445_MOESM11_ESM.pdf]

Suppl. 11: Bacterial composition significantly differ between mosquitoes exposed to different insecticides.

a.

Group 1	Group 2	N	pseudo-F	p-value	q-value
**Alphacypermethrin**	**Deltamethrin**	**67**	**17.42**	**0.001**	**0.002**
**Alphacypermethrin**	**None**	**60**	**10.26**	**0.001**	**0.002**
**Alphacypermethrin**	**Permethrin**	**96**	**10.10**	**0.001**	**0.002**
**Deltamethrin**	**None**	**79**	**3.13**	**0.017**	**0.026**
Deltamethrin	Permethrin	115	2.11	0.073	0.073
**None**	**Permethrin**	**108**	**2.43**	**0.028**	**0.034**

b.

			Internal microbiota				Cuticle surface microbiota			
Stage	Group 1	Group 2	N	pseudo-F	p-value	q-value	N	pseudo-F	p-value	q-value
Adult	Alphacypermethrin	Deltamethrin	18	1.43	0.222	0.222	18	1.28	0.25	0.375
	**Alphacypermethrin**	**None**	**21**	**7.54**	**0.001**	**0.003**	**21**	**2.78**	**0.005**	**0.03**
	Alphacypermethrin	Permethrin	30	2.13	0.057	0.068	30	1.75	0.123	0.369
	**Deltamethrin**	**None**	**15**	**2.78**	**0.029**	**0.044**	15	1.34	0.2	0.375
	**Deltamethrin**	**Permethrin**	**24**	**5.65**	**0.002**	**0.004**	24	0.83	0.494	0.593
	**None**	**Permethrin**	**27**	**16.41**	**0.001**	**0.003**	27	0.74	0.619	0.619
Larvae	**Deltamethrin**	**None**	**26**	1.91	0.075	0.225	23	**3.26**	**0.016**	**0.024**
	Deltamethrin	Permethrin	35	0.86	0.514	0.514	32	1.03	0.365	0.365
	**None**	**Permethrin**	**27**	1.44	0.173	0.26	27	**3.83**	**0.002**	**0.006**

Pair-wise comparisons of beta diversity (Bray Curtis) between groups of *An. albimanus* that were exposed to alphacypermethrin, deltamethrin or permethrin overall (a) and by developmental stage (b) show that bacterial composition differ significantly between mosquitoes exposed to different insecticides. Comparisons were conducted using PERMANOVA (999 permutations) tests with Benjamini-Hochberg FDR correction (q-value). Significance is set to q-value (adjusted p-value) <0.05. N = number of mosquito pool included in the analysis; each pool comprised 3 mosquitoes.
