# Supplementary material for: Pyrethroid exposure alters internal and cuticle surface bacterial communities in Anopheles albimanus
Source: ISME J. 2019 Jun 6;13(10):2447–64. doi: 10.1038/s41396-019-0445-5 (PMC6776023; doi:10.1038/s41396-019-0445-5)

Suppl. 14. Log ratios of ASVs in gneiss balance y0, and number of unique taxa (at the species level) that contributed to shifts in bacterial composition between insecticide exposed and non-exposed mosquitoes.

The boxplots show the distribution of log-transformed ratios of ASV abundance between insecticide exposed and non-exposed mosquitoes. The top unique taxa in y0 numerator and denominator that contributed to the shift in community composition between groups are shown in the bar plots to the right.

Adult microbiota

Internal: Alphacypermethrin

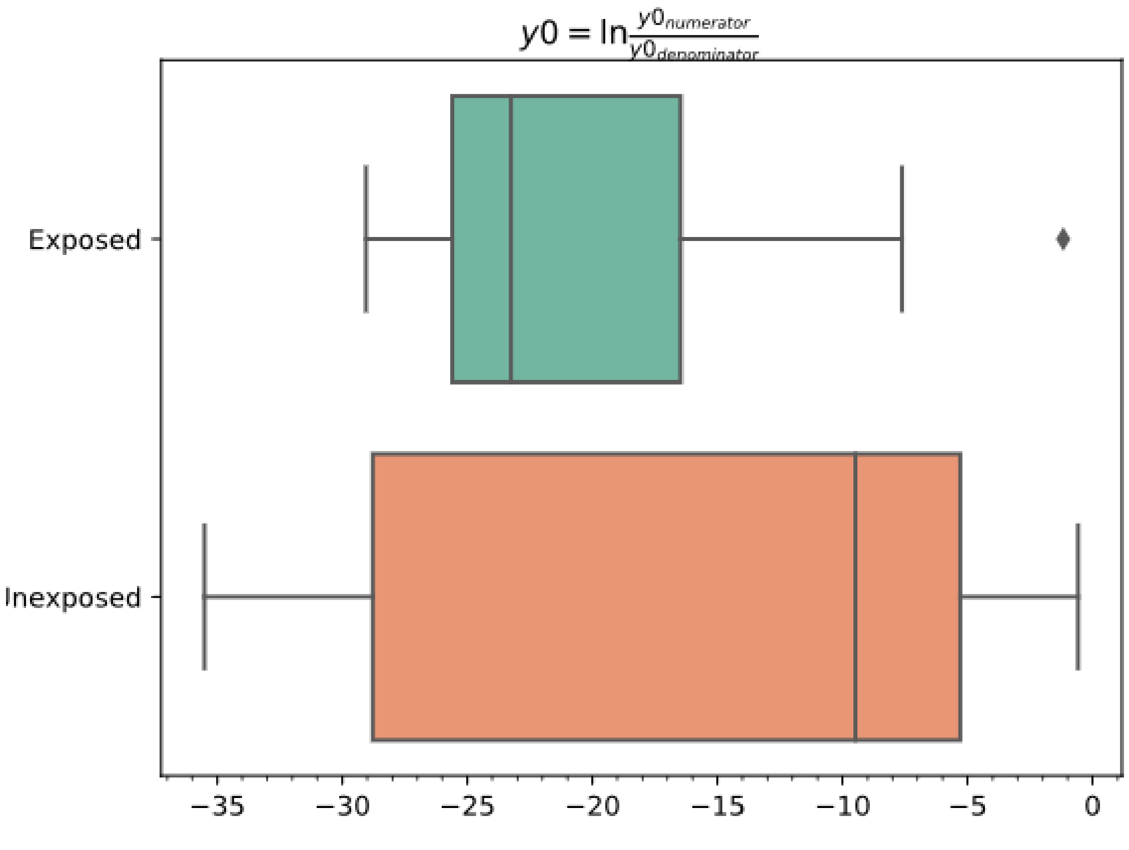

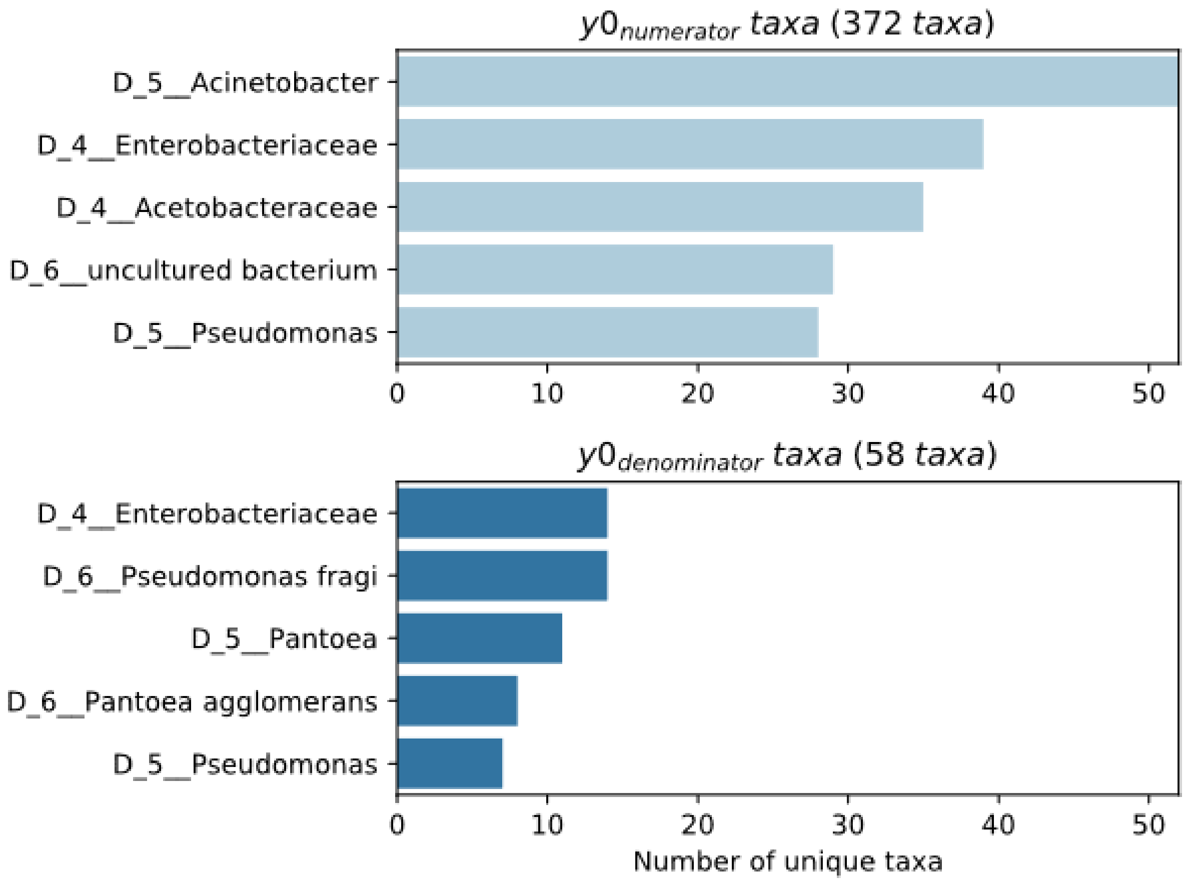

Internal: Permethrin

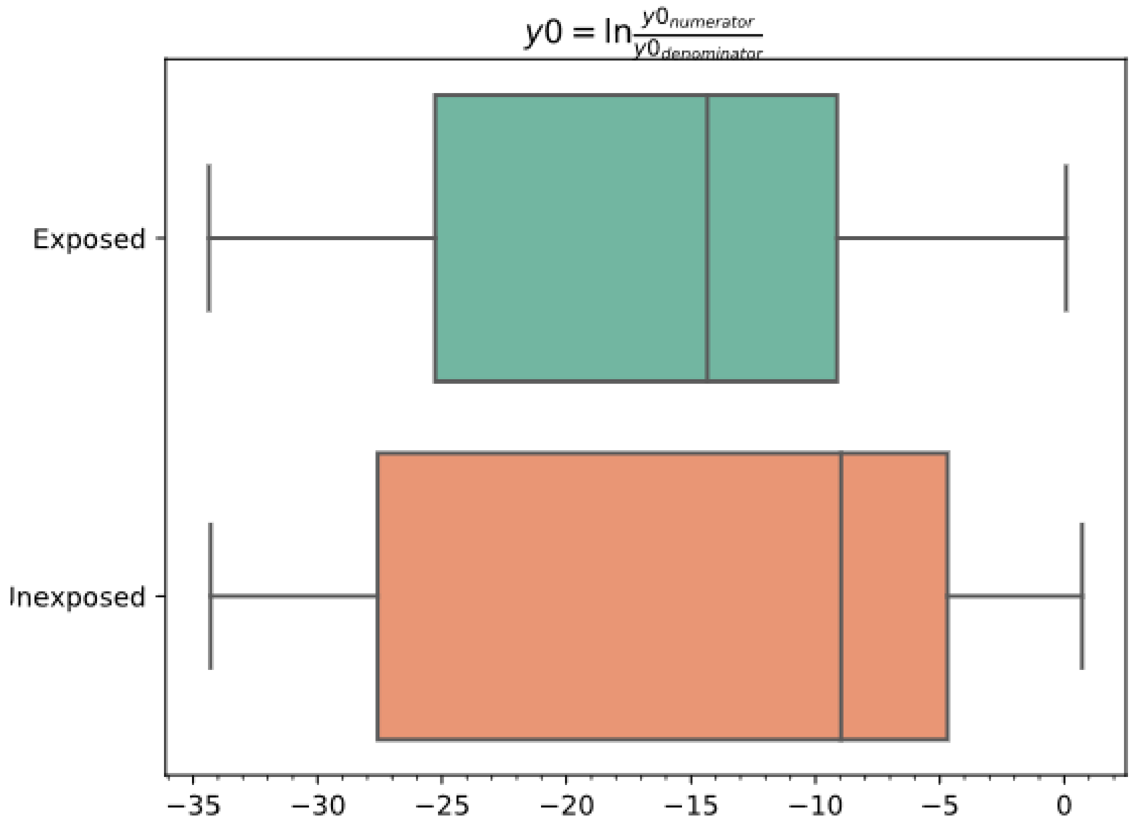

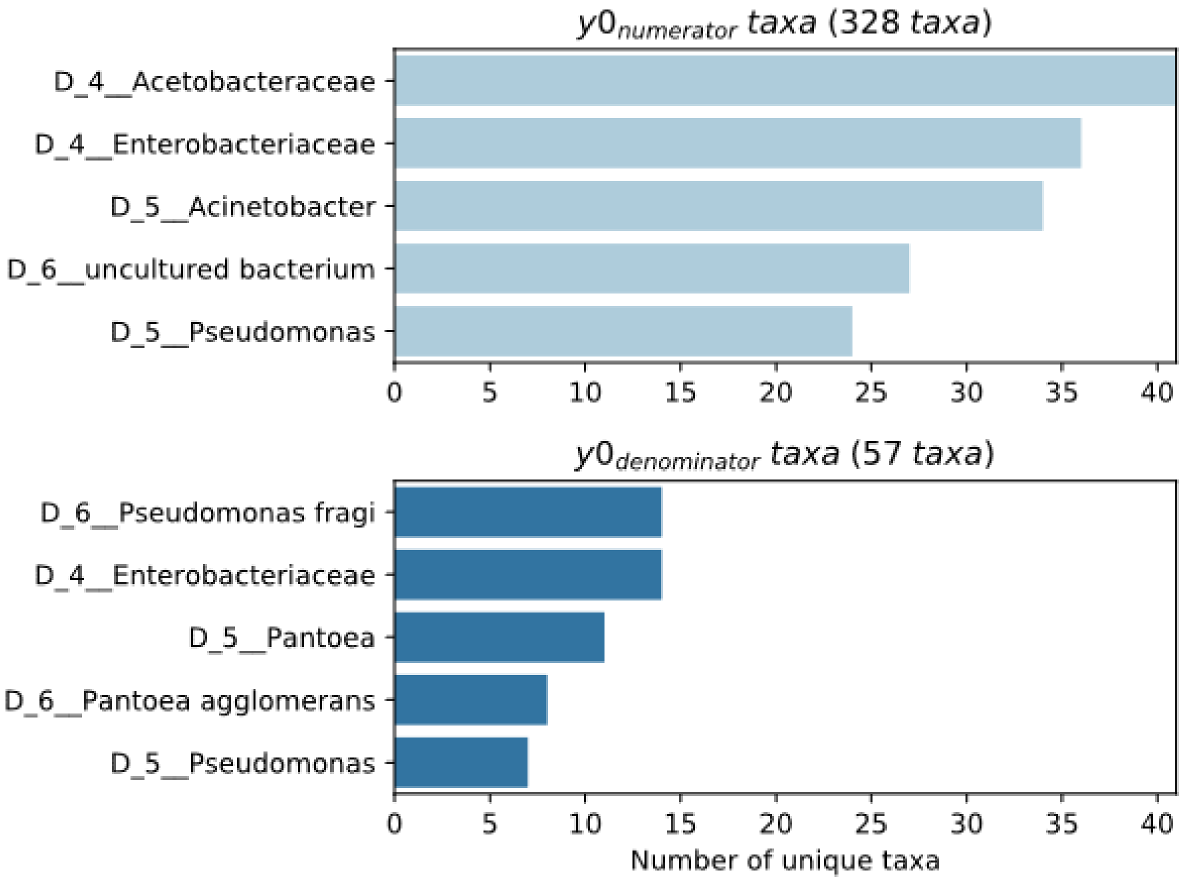

Cuticle surface: Alphacypermethrin

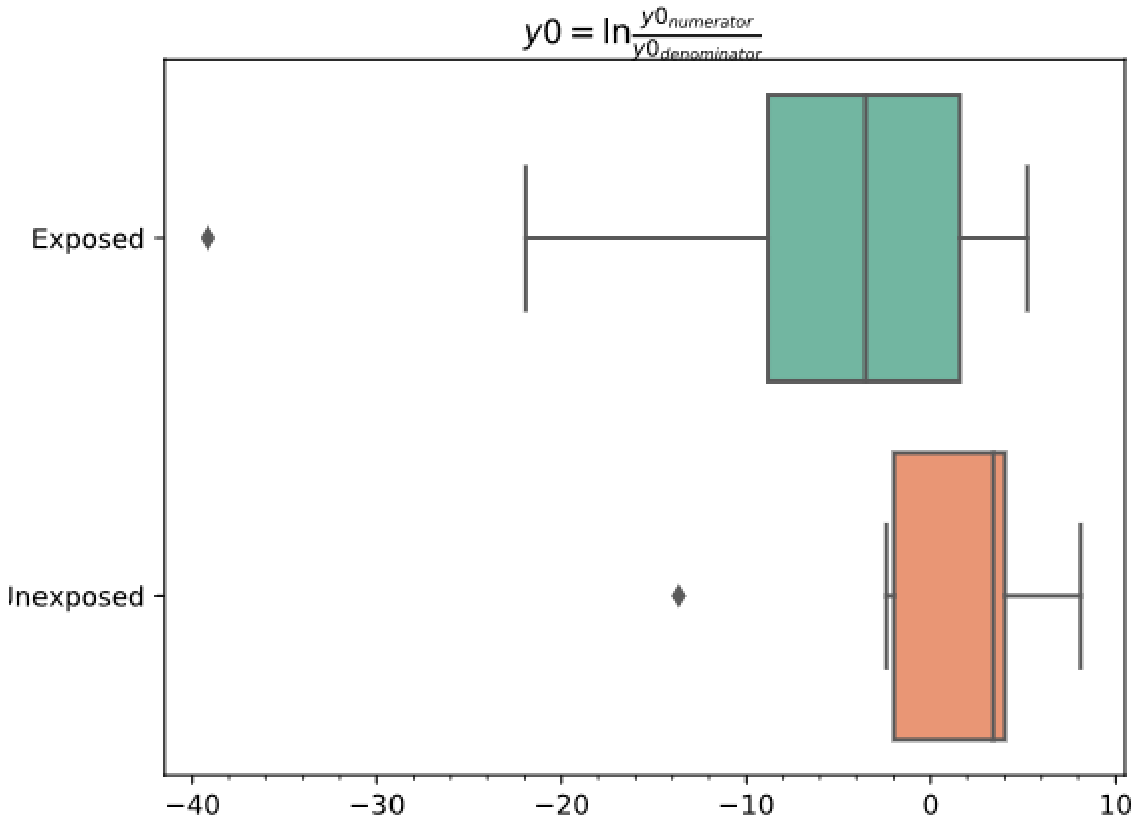

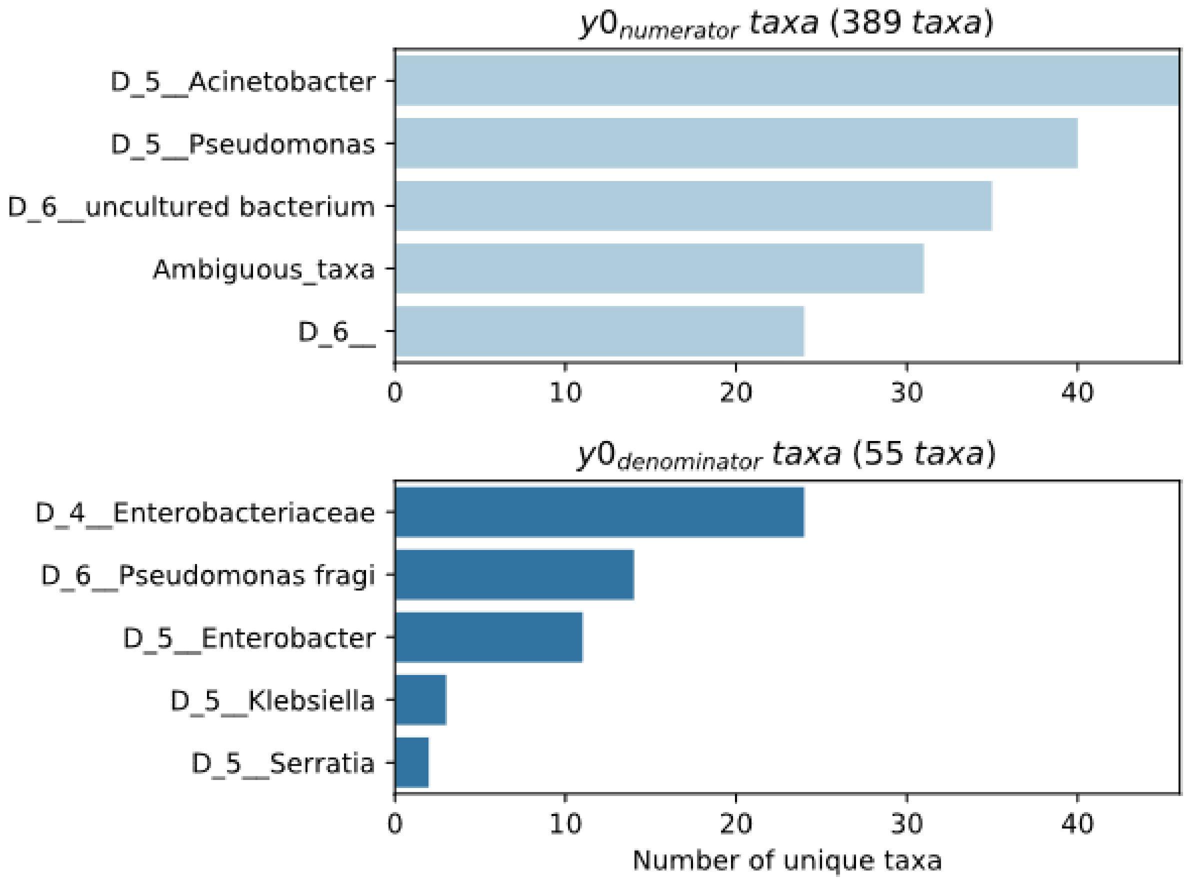

Larval microbiota

Cuticle surface: Deltamethrin

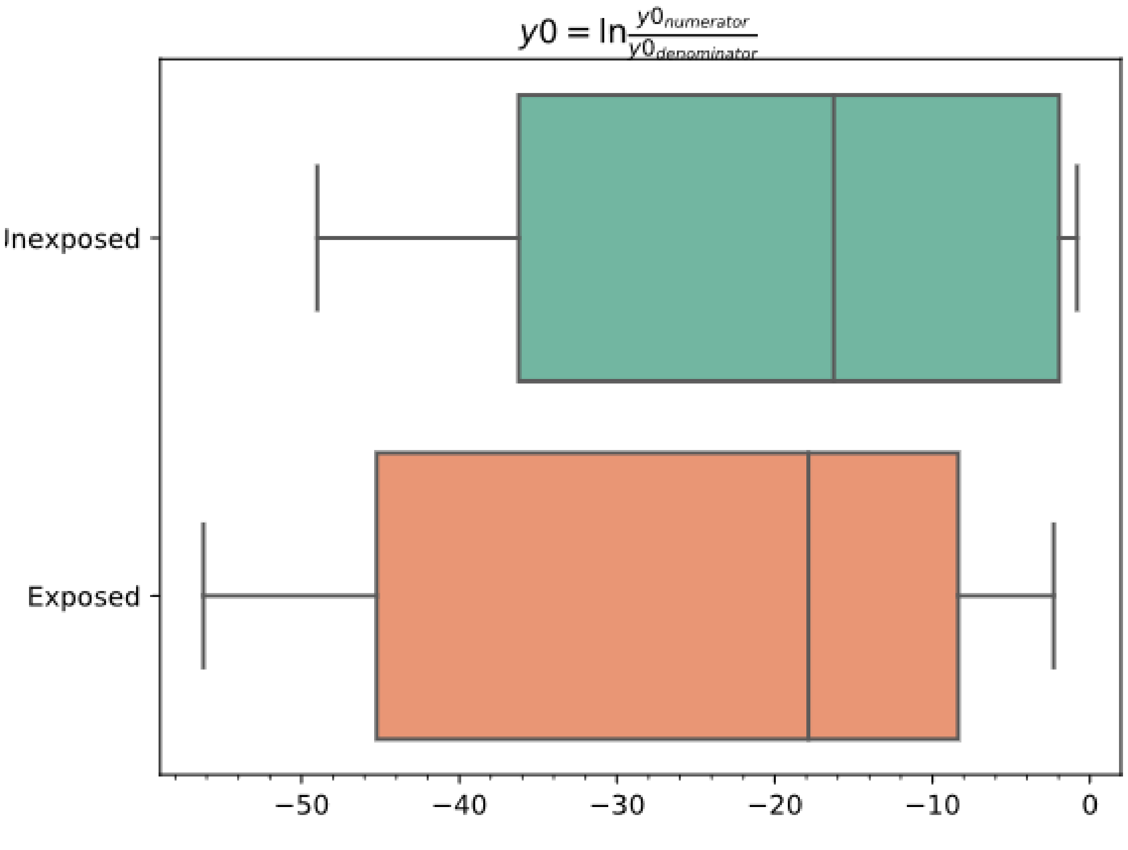

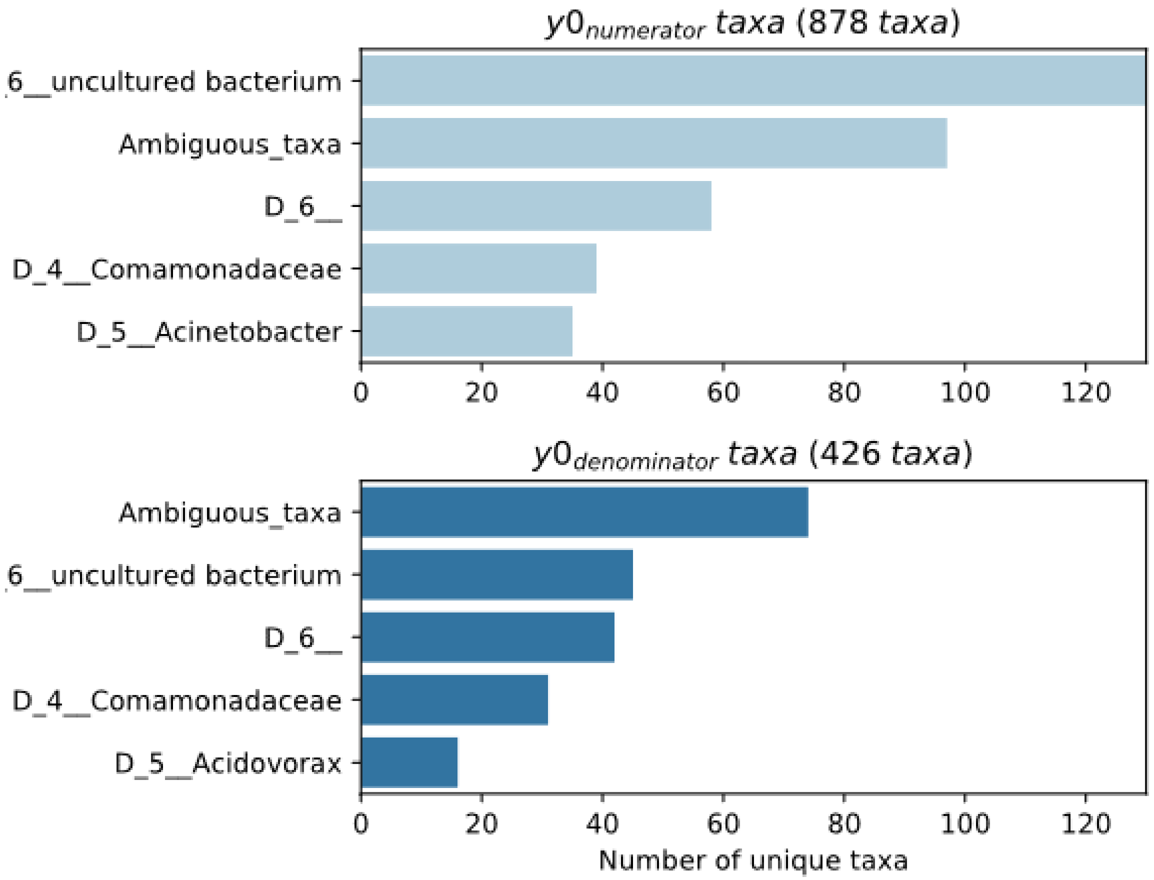

Cuticle surface: Permethrin

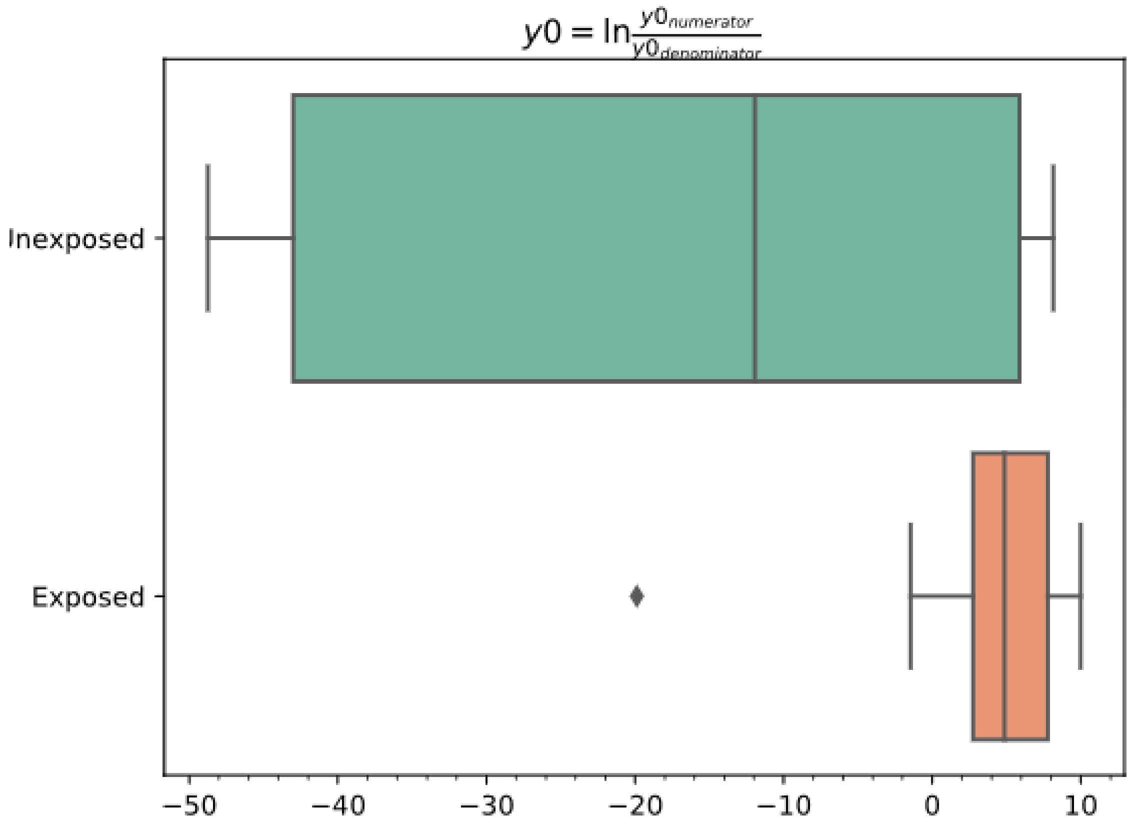

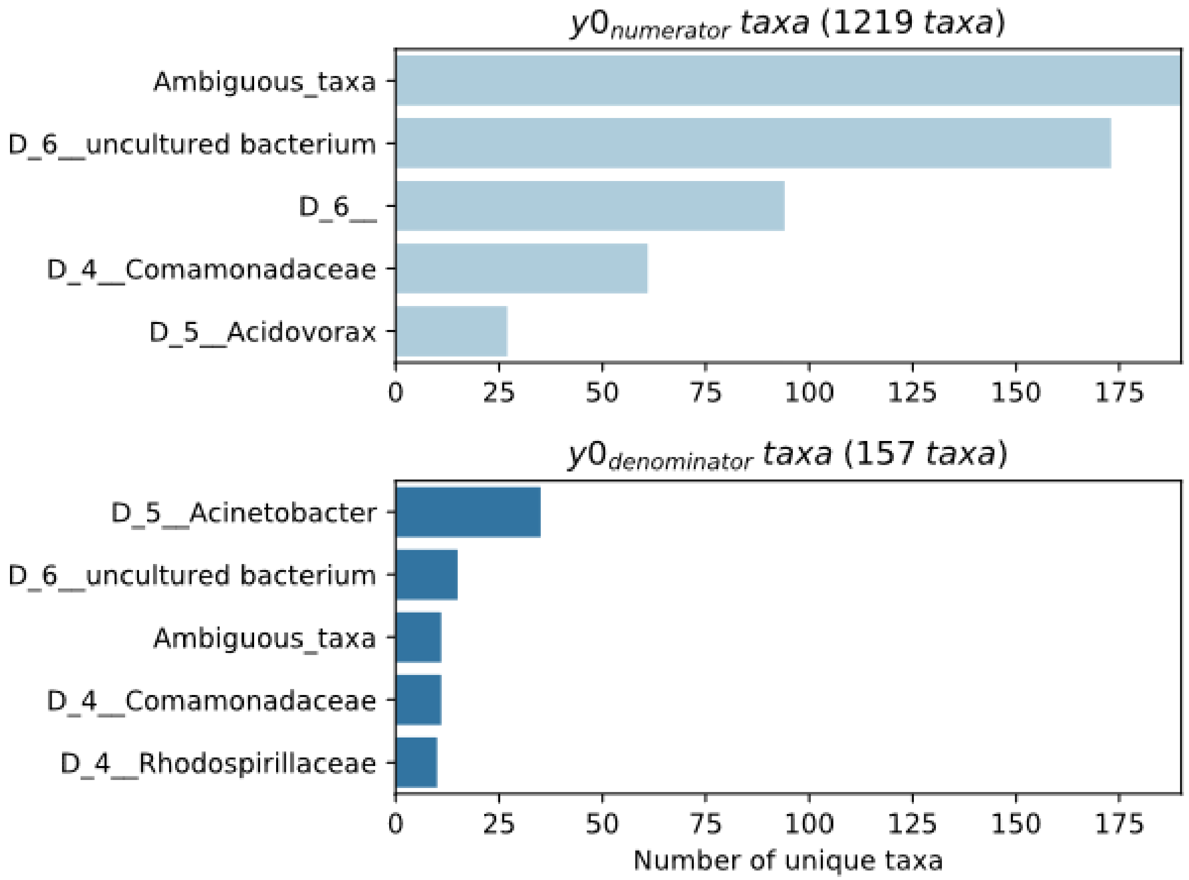

Supplement: Supplementary file 14 — Suppl. 14 [file 41396_2019_445_MOESM14_ESM.pdf]
